# OpenAM-SimCCX: An Open-Source Framework for Thermo-Mechanical Analysis of Additive Manufacturing with CalculiX

**DOI:** 10.3390/ma18214990

**Published:** 2025-10-31

**Authors:** Jesus Romero-Hdz, Baidya Nath Saha, Jobish Vallikavungal, Patricia Zambrano-Robledo

**Affiliations:** 1 Facultad de Ingeniería Mecánica y Eléctrica, Universidad Autónoma de Nuevo León, San Nicolás de los Garza 66455, Mexico; jr90236@gmail.com; 2 Department of Mathematics and Information Technology, Concordia University of Edmonton, Ada Blvd NW, Edmonton, AB T5B 4E4, Canada; baidya.saha@concordia.ab.ca; 3 School of Engineering and Sciences, Tecnológico de Monterrey, Monterrey 64849, Mexico; jobish03@tec.mx

**Keywords:** additive manufacturing, selective laser sintering, CalculiX, thermo-mechanical simulation, residual stress, deformation prediction, layer-by-layer element activation, laser–material interaction, scanning strategies, finite element analysis, open-source framework, computational efficiency

## Abstract

Additive Manufacturing (AM) has emerged as a transformative technology for rapid prototyping and fabrication of geometrically complex structures. However, the inherent thermal cycling and rapid solidification in processes such as Selective Laser Sintering (SLS) frequently induce deformation and residual stresses, leading to dimensional deviations and potential premature failure. This paper presents OpenAM-SimCCX, an open-source workflow for finite element-based thermo-mechanical simulation of AM processes using CalculiX 2.21. The framework employs a time-dependent thermo-mechanical model with layer-by-layer element activation to capture key aspects of SLS, including laser–material interaction and scanning strategy effects. Systematic comparisons of different scanning strategies demonstrate clear correlations between path planning, residual stress distributions, and distortion, while computational time analyses confirm the framework’s efficiency. By providing comprehensive documentation, implementation guides, and open repositories, OpenAM-SimCCX offers an accessible and economically viable alternative to commercial software, particularly for academic institutions and small- to medium-sized enterprises. This framework advances open-source simulation tools for AM and promotes broader adoption in both research and industry.

## 1. Introduction

Additive Manufacturing (AM) has fundamentally transformed modern manufacturing by enabling rapid prototyping and the fabrication of geometrically intricate structures that are difficult or impossible to achieve through conventional methods [1,2]. Among various AM technologies, Selective Laser Sintering (SLS) has emerged as a particularly prominent technique due to its ability to produce durable components directly from powdered materials. The SLS process offers significant advantages, including design flexibility, reduced material waste, and rapid production capabilities, which makes it attractive for aerospace, automotive, biomedical, and consumer product applications [3].

Despite these advantages, the sophisticated thermal processes inherent in SLS, characterized by localized heating, steep thermal gradients, and rapid cooling, introduce major challenges. These include the generation of residual stresses, distortions, and dimensional deviations that can compromise the structural integrity, reliability, and functionality of manufactured parts [4,5]. The complex interplay of thermal gradients, material properties, and process parameters creates a challenging optimization problem that requires advanced predictive tools to address.

Numerical simulation has emerged as a powerful approach to analyze and optimize AM processes by providing insight into heat transfer, phase transformations, and mechanical deformation, thus reducing the need for costly empirical testing [6,7]. However, the adoption of such advanced simulation methods is often constrained by the prohibitive cost of commercial software and the specialized hardware it requires, which limits the accessibility for academic institutions, researchers, and small to medium-sized enterprises (SMEs).

To address these challenges, this paper presents OpenAM-SimCCX, a comprehensive and cost-effective workflow for thermo-mechanical simulation of selective laser sintering (SLS) processes using the open-source finite element solver CalculiX. The proposed methodology couples a time-dependent thermo-mechanical model with advanced modeling features, including Gaussian heat source representation, layer-by-layer element activation, and temperature-dependent material properties [8,9,10,11]. This integrated framework enables accurate prediction of deformation and provides valuable insights into final part quality.

To ensure reliability, the proposed workflow is compared with reference cases from the established literature, with systematic evaluations of scanning strategies, deformation patterns, and residual stress distributions. By combining predictive accuracy with computational efficiency, OpenAM-SimCCX offers capabilities comparable to commercial solutions while remaining fully accessible as an open-source framework.

Finally, recognizing the importance of community-driven development, this research provides extensive documentation, preprocessing scripts, material property databases, and open-source repositories. These resources are designed to facilitate reproducibility, encourage collaboration, and promote the broader adoption of AM simulation practices within both the academic and industrial communities.

## 2. Literature Review

This literature review commences by exploring various simulation approaches and their applications in additive manufacturing.

### 2.1. Simulation Approaches in Additive Manufacturing

Numerical simulation has become a cornerstone in analyzing and optimizing metal Additive Manufacturing (AM) processes, particularly Laser Powder Bed Fusion (LPBF). These processes are governed by intense, localized thermal cycling that produces steep thermal gradients, residual stresses, and geometric distortions, which directly impact part quality and performance. Traditional analytical approaches are insufficient to capture such multiphysics phenomena, making simulation indispensable.

Finite Element Analysis (FEA) has emerged as the dominant methodology for modeling thermo-mechanical behavior in AM. Modern FEA-based approaches typically employ a sequentially coupled scheme: first, transient heat transfer is modeled to establish the thermal history; subsequently, the resulting temperature fields are used to drive mechanical stress and deformation analysis [8]. This coupling strategy balances computational efficiency with physical fidelity, enabling the simulation of layer-by-layer manufacturing over complex geometries.

Recent studies have demonstrated the effectiveness of this approach. For instance, Poyraz et al. [12] employed Simufact Additive to predict residual stresses in LPBF-fabricated Inconel 625 parts, showing the importance of accurate material data and realistic boundary conditions in achieving reliable predictions. Commercial tools such as ABAQUS and Simufact Additive remain widely adopted due to their robust solvers and pre-integrated AM modules. ABAQUS, for example, has supported multi-scale thermo-mechanical simulations through progressive element activation and user subroutines, as demonstrated by Balbaa and Elbestawi [13] and Yang et al. [14]. Simufact Additive further facilitates voxel-based meshing, inherent strain modeling, and includes material databases tailored for LPBF applications.

Despite their strengths, commercial software often functions as a black box, limiting transparency and user control. In contrast, custom solvers such as AdhoC++ 2022 an in-house finite element framework continuously developed and maintained at the Technical University of Munich (TUM)  [15] provide full flexibility for implementing bespoke heat transfer and material behavior models, though at the cost of significant programming effort and extensive validation. Open-source alternatives such as CalculiX [11] strike a practical balance: while lacking dedicated AM modules and advanced GUIs, CalculiX offers extensibility via user subroutines and Python based preprocessing/postprocessing workflows. These capabilities allow researchers to model phenomena such as evolving porosity, temperature-driven phase transformations, and path-dependent scanning strategies in a cost-effective manner.

To address the computational expense of high-fidelity AM simulations, advanced strategies have been introduced. Huang et al. [16] developed a GPU-accelerated explicit FEA framework that grouped tracks and layers, achieving more than 50-fold speedup compared to conventional ABAQUS implementations, while maintaining predictive accuracy for residual stress and distortion.

In summary, simulation approaches in AM have evolved from basic thermo-mechanical models to advanced, customizable frameworks that balance accuracy, efficiency, and accessibility. While commercial software remains dominant in industrial practice, open-source platforms such as CalculiX increasingly provide powerful, transparent, and economically viable alternatives for researchers and SMEs.

### 2.2. Limitations of Commercial Tools

Commercial FEA software, including ABAQUS, Simufact Additive, Amphyon, and Netfabb, offers advanced capabilities for simulating Additive Manufacturing (AM) processes. These platforms provide robust numerical solvers, pre-integrated AM modules, and extensive material libraries, enabling complex multi-physics simulations. However, several limitations constrain their utility, particularly in research, exploratory studies, and resource-constrained environments.

The primary concern is the internal numerical formulations. As noted by Poyraz et al. [12], commercial platforms often obscure critical assumptions regarding heat input, material interpolation, and solver stabilization. This opacity complicates rigorous model validation, limits reproducibility, and restricts the extension to non-standard materials or novel physical effects. Furthermore, many commercial tools rely on the inherent strain method for residual stress prediction, which, while computationally efficient, exhibits limited generalizability. Bugatti and Semeraro [17] demonstrated that this approach is accurate only when carefully calibrated for specific geometries, and its extrapolation to new configurations can introduce significant errors. Important time-dependent phenomena, such as stress relaxation and creep, are frequently neglected, reducing accuracy for components with complex thermal histories [18].

The customization limitations of commercial platforms further impede advanced research. Although user-defined subroutines (e.g., UMAT, DFLUX, HETVAL) are supported, access to core solver functionality remains restricted. Researchers, such as Yang et al. [14] and Balbaa and Elbestawi [13], have had to implement extensive preprocessing and scripting workflows to simulate realistic laser scanning patterns and material activation sequences, highlighting the limited flexibility and control available to users.

Computational cost is another critical constraint. Implicit solvers, commonly used in commercial software, can become prohibitively slow for high-resolution or large-scale models. Huang et al. [16] addressed this by developing a GPU-based explicit solver, which significantly accelerated computations without sacrificing predictive accuracy. Beyond computational demands, the high licensing costs of commercial platforms restrict accessibility for academic institutions, small enterprises, and budget-constrained research groups. Many commercial tools also lack integrated optimization capabilities, such as automated build orientation or support structure design, requiring users to develop their own routines [19].

In summary, while commercial FEA software provides powerful tools for AM simulation, these platforms face challenges related to transparency, flexibility, computational efficiency, and cost. These limitations underscore the need for alternative frameworks particularly open-source solutions that enable detailed, customizable, and cost-effective modeling of AM processes for research and industrial applications.

### 2.3. Open-Source Alternatives and the Role of CalculiX

Open-source finite element platforms provide a viable alternative to commercial tools, particularly for research applications requiring full control over the solver logic, process customization, and methodological transparency. Among these platforms, CalculiX has emerged as a promising solution due to its extensibility, robust numerical foundation, and support for user-defined subroutines, allowing researchers to implement advanced material models, specialized boundary conditions, and custom heat source formulations.

Silva et al. [20] demonstrated the capabilities of CalculiX in simulating Selective Laser Sintering (SLS) for Inconel 625. Their study incorporated a Gaussian heat source and porosity evolution, predicting peak residual stresses of 230 MPa and part distortions up to 0.027 mm. Although experimental validation was not conducted, the results highlighted the feasibility of advanced material modeling within an open-source framework and underscored the potential of CalculiX for high-fidelity AM simulations.

Building on this foundation, Goetz et al. [21] developed AscentAM, a modular simulation framework based on CalculiX. The framework supports sequential thermo-mechanical analysis, layer-wise material activation, comprehensive process chain modeling, and uncertainty quantification. Validation against high-resolution 3D scan data demonstrated dimensional accuracies up to 94.7% and average geometric deviations below 0.2 mm, confirming the industrial applicability of open-source FEA workflows.

Additionally, Dreibati [11] illustrated the flexibility of CalculiX implementing layer-by-layer simulation strategies. This methodology accurately models the SLS process, including powder spreading, laser beam trajectory, and element activation sequences, enabling detailed analysis of temperature evolution while maintaining computational efficiency via selective mesh refinement and element activation.

Collectively, these studies show that with careful extensions, validation, and methodological rigor, CalculiX can serve as a robust and cost-effective platform for high-fidelity AM simulations. Its open-source nature provides a transparent and flexible alternative to commercial software, empowering researchers and small enterprises to explore novel material models, complex heat transfer effects, and advanced process simulations that would be challenging to achieve in proprietary tools.

While open-source simulation environments have significantly improved the accessibility and transparency of additive manufacturing research, most traditional frameworks remain limited by CPU-bound computational performance. To address these constraints, recent efforts have focused on leveraging Graphics Processing Units (GPUs) to accelerate numerical solvers and enable high-fidelity, large-scale simulations. These emerging GPU-enhanced frameworks represent the next evolutionary step in open and extensible AM modeling platforms.

### 2.4. GPU-Accelerated and Open-Source Frameworks

Recent advancements in high-performance computing have driven the development of GPU-accelerated frameworks tailored for additive manufacturing (AM). These frameworks significantly reduce simulation times while maintaining high fidelity in thermomechanical modeling. For instance, Huang et al. [16] presented an open-source GPU-based solver optimized for layer-wise thermal field prediction, demonstrating near real-time computation capabilities compared to CPU-based alternatives. Similarly, Liao et al. [22] developed a GPU-accelerated thermomechanical solver capable of accurately predicting residual stresses in metal AM parts, providing a foundation for multi-physics process modeling.

The emergence of specialized frameworks such as [21] (AscentAM) and [23] (AdhoC++) reflects a growing trend toward community-driven and modular simulation architectures. These tools integrate open-source numerical kernels with CUDA or HIP backends, allowing researchers to extend functionalities for specific AM modalities or materials without the constraints of proprietary software.

In parallel, Luthi et al. [24] introduced MULTI-3, a GPU-enhanced meshfree simulation framework designed for multi-track, multi-layer, and multi-material laser powder bed fusion (LPBF) processes. The framework demonstrates scalability across multiple GPUs and captures the complex thermal interactions between overlapping melt pools. Collectively, these developments illustrate how open-source, GPU-accelerated solvers are reshaping the computational landscape of additive manufacturing research by balancing accuracy, scalability, and accessibility.

### 2.5. Experimental Validation and Realism

Despite the sophistication of contemporary thermo-mechanical simulation methods for metal additive manufacturing (AM), rigorous experimental validation remains essential to ensure predictive realism, establish model credibility, and build confidence in simulation-based design decisions. The inherent complexity of AM processes, involving coupled thermal, mechanical, and metallurgical phenomena, necessitates validation against high-fidelity experimental measurements to confirm the accuracy and reliability of simulation predictions.

Common validation techniques include X-ray diffraction (XRD) for residual stress measurement, high-resolution 3D optical scanning for geometric accuracy assessment, blind-hole drilling for localized stress evaluation, and advanced microscopy for microstructural characterization. These complementary methods enable comprehensive evaluation of simulation predictions across multiple scales and physical effects.

Recent studies have provided quantitative insights into the effectiveness of validation approaches. For example, Sikan et al. [25] reported residual stress prediction deviations of up to 100 MPa via XRD measurements, highlighting areas where simulation models require refinement. Conversely, Singh et al. [26] demonstrated improved agreement with experimental data, reporting stress prediction errors within 7% through careful calibration of material properties and boundary conditions.

Geometric accuracy has also been systematically assessed. Sikan et al. achieved high-fidelity distortion predictions with errors below 0.05 mm using ATOS 3D scanning systems. Similarly, Bayat et al. [27] showed that refined thermal boundary condition modeling can reduce distortion prediction errors from 46.2% to 1.19%, emphasizing the critical importance of accurate physical modeling in achieving realistic simulation outcomes.

A key limitation remains: the majority of validated studies to date have relied on commercial FEA software platforms. Open-source solvers, such as CalculiX, have not yet undergone systematic experimental validation, which limits their adoption in high-fidelity AM research and industrial applications. Addressing this gap represents an important priority for future studies, enabling open-source tools to achieve the same credibility and predictive power as commercial alternatives.

In summary, experimental validation is indispensable for confirming simulation fidelity in AM, providing the necessary foundation for both academic research and industrial process optimization.

### 2.6. Scan Strategies and Their Effects

Scan strategy is a critical process parameter in powder bed fusion (PBF) additive manufacturing, directly influencing thermal gradients, residual stress distributions, microstructural evolution, and final part distortion. Both experimental and computational studies consistently show that careful planning of scan paths can significantly affect stress anisotropy, melt pool stability, and geometric accuracy of fabricated components.

Alternating and rotational scanning strategies, such as interlayer hatch rotations of $45^{\circ }$, $67^{\circ }$, and $90^{\circ }$, have been shown to reduce residual stresses and improve part quality. Paraschiv et al. [28] demonstrated that $90^{\circ }$ chessboard and strip scanning patterns decreased post-processing distortion by 39–63%. Similarly, Monu et al. [29] reported reductions in normalized von Mises stress from 0.84 to 0.13 using bidirectional and rotational scanning strategies.

Inclined scanning strategies, for example $45^{\circ }$ hatching, also improve heat distribution and control stress accumulation. Cheng et al. [30] observed reduced build-direction warping in samples using $45^{\circ }$ scanning patterns. Zhang et al. [31] and Carraturo et al. [15] reported enhanced residual stress uniformity and melt pool stability in AlSi10Mg and IN625, respectively, under inclined scanning approaches.

Advanced scanning strategies, including dual-laser time-delayed scanning [32] and subdivided hatching [27], have further improved simulation fidelity and predictive accuracy. Flash heating techniques, in particular, have been shown to reduce distortion error from 46.2% to 1.19%, as verified through high-resolution 3D scanning.

Despite these advancements, most scan strategy simulations are implemented using commercial software, limiting accessibility for experimental and exploratory research. Open-source platforms, such as CalculiX, remain underutilized, though they offer substantial potential for custom scan path modeling, advanced analyses, and broader dissemination within the research community.

In summary, optimized scan strategies are pivotal in controlling thermal and mechanical phenomena during PBF, directly impacting residual stress, distortion, and part quality. The combination of experimental validation and advanced simulation enables both the refinement of scanning techniques and the development of robust, predictive models for additive manufacturing.

### 2.7. Summary and Research Gap

The literature on metal additive manufacturing (AM) highlights substantial progress in simulating residual stress and distortion, particularly through finite element analysis (FEA) in laser powder bed fusion (LPBF) processes. Commercial software platforms provide well-integrated AM modules and comprehensive material libraries, enabling robust simulation capabilities. However, these tools exhibit several notable limitations: they restrict solver customization, rely on simplified modeling approaches such as inherent strain methods, and require expensive licenses, which limits accessibility and reduces transparency and generalizability of the results.

Open-source solvers, such as CalculiX, offer distinct advantages in flexibility, extensibility, and cost-effectiveness. Emerging studies demonstrate their potential for accurate thermo-mechanical simulation of AM processes, including complex material behavior and sequential layer-wise modeling. Despite these advantages, open-source platforms remain under-validated, especially for simulations that incorporate realistic scanning strategies, material activation sequences, and practical boundary conditions.

A significant gap persists in the current research landscape: the absence of comprehensive, experimentally validated, open-source thermo-mechanical simulation workflows that integrate detailed scan path modeling, realistic material properties, and physically representative boundary conditions. Addressing this gap is critical for enabling high-fidelity AM simulations accessible to both the research and industrial communities.

This work directly addresses this gap by developing a CalculiX based simulation pipeline that enables sequential thermal and mechanical analysis using validated input parameters and realistic scanning strategies. The primary objectives of this research are to:

- Provide a fully accessible, open-source framework for high-fidelity AM simulation.
- Incorporate detailed scan path modeling and advanced material activation schemes.
- Validate the simulation workflow against experimental data to ensure predictive realism.

By achieving these objectives, this research advances the state of metal AM simulation, offering a transparent, customizable, and experimentally grounded alternative to proprietary software. This framework lays the foundation for subsequent methodological development and practical application in additive manufacturing.

## 3. Layer-by-Layer Thermo–Mechanical Finite Element Method for Additive Manufacturing

This section presents a unified finite element (FE) framework for simulating the layer-by-layer thermo–mechanical behavior in metal additive manufacturing (AM). The formulation proceeds as follows:

(a) Define the governing PDEs for transient heat conduction and quasi-static thermoelasticity.
(b) Derive the corresponding weak forms.
(c) Formulate element-level matrices and load vectors (mass, stiffness, and thermal components).
(d) Specify quadrature and numerical integration schemes.
(e) Describe assembly, boundary condition enforcement, time integration, and coupling strategies.
(f) Conclude with a practical algorithmic workflow for implementation and reproducibility.

### 3.1. Problem Statement and Strong Form

Consider a part domain $\Omega \subset R^{3}$ with boundary $\partial \Omega =\Gamma_{D}\cup \Gamma_{N}$. The coupled thermo–mechanical problem is governed by two partial differential equations (PDEs).

(a) Thermal (Transient Heat Conduction):

(1) $$\rho \left(T\right)\;c_{p}\left(T\right)\;\frac{\partial T}{\partial t}-\nabla \;\cdot \;\left(k(T)\nabla T\right)=Q\left(x,t\right)$$

subject to

(2) $$-k\left(T\right)\nabla T\cdot n=\bar{q}\;\mathrm{on}\;\Gamma_{q},\;T=\bar{T}\;\mathrm{on}\;\Gamma_{D},\;T\left(x,0\right)=T_{0}\left(x\right).$$

(b) Mechanical (Quasi-Static Equilibrium):

(3) ∇·σ(U,T)+b=0,

with

(4) $$\sigma \cdot n=\bar{t}\;\mathrm{on}\;\Gamma_{t},\;U=\bar{U}\;\mathrm{on}\;\Gamma_{U}.$$

### 3.2. Weak (Variational) Forms

Thermal Weak Form. Multiplying the heat equation by a test function $w\in H^{1}\left(\Omega \right)$ and integrating by parts gives:

(5) $$\int_{\Omega }\rho c_{p}\dot{T}w\;d\Omega +\int_{\Omega }k\nabla T\cdot \nabla w\;d\Omega =\int_{\Omega }Qw\;d\Omega +\int_{\Gamma_{q}}\bar{q}w\;d\Gamma .$$

In AM, *Q* or $\bar{q}$ is often modeled as a Gaussian surface heat flux:

(6) $$q\left(x,y,z,t\right)=\frac{2\eta P}{\pi R^{2}}\exp\;\left(-\frac{2r^{2}}{R^{2}}\right),$$

where *P* is laser power, η absorption efficiency, *R* beam radius, and *r* the radial distance from the beam center. This expression contributes directly to the FE thermal load vector $F_{q}$.

**Figure A1:**
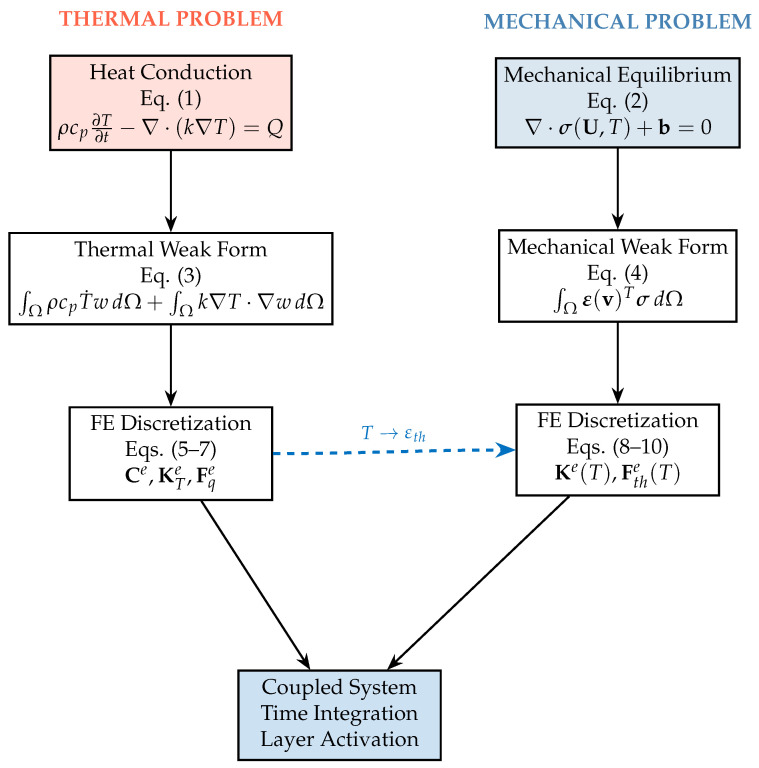
Workflow connecting governing equations to finite element implementation in additive manufacturing simulation. Thermal analysis drives mechanical response through thermal strain coupling ($\varepsilon_{th}=\alpha \left(T-T_{ref}\right)$), capturing residual stress generation during layer-by-layer deposition.

Mechanical Weak Form. For a test function $v\in {\left[H^{1}\left(\Omega \right)\right]}^{3}$ vanishing on $\Gamma_{U}$:

(7) $$\int_{\Omega }\varepsilon {\left(v\right)}^{T}\sigma \;d\Omega =\int_{\Omega }v\cdot b\;d\Omega +\int_{\Gamma_{t}}v\cdot \bar{t}\;d\Gamma ,$$

where $\varepsilon \left(v\right)=\frac{1}{2}\left(\nabla v+\nabla v^{T}\right)$. The coupling arises from temperature-dependent material properties and thermal strain, allowing accurate prediction of residual stresses and distortions (see Figure A1).

### 3.3. Finite Element Discretization

Let $V_{h}\subset H^{1}\left(\Omega \right)$ and $V_{h}\subset {\left[H^{1}\left(\Omega \right)\right]}^{3}$ denote the FE spaces for *T* and U, respectively. Isoparametric 8-node hexahedral elements with trilinear interpolation are used.

Thermal Matrices:

(8) $$\begin{array}{cc} C_{ij}^{e} & =\int_{\Omega^{e}}\rho c_{p}\phi_{i}\phi_{j}\;d\Omega , \end{array}$$

(9) $$\begin{array}{cc} K_{T,ij}^{e} & =\int_{\Omega^{e}}k\nabla \phi_{i}\cdot \nabla \phi_{j}\;d\Omega , \end{array}$$

(10) $$\begin{array}{cc} F_{q,i}^{e} & =\int_{\Omega^{e}}Q\phi_{i}\;d\Omega +\int_{\partial \Omega_{q}^{e}}\bar{q}\;\phi_{i}\;d\Gamma . \end{array}$$

Mechanical Matrices:

(11) $$\begin{array}{cc} K^{e}\left(T\right) & =\int_{\Omega^{e}}B^{T}D\left(T\right)B\;d\Omega , \end{array}$$

(12) $$\begin{array}{cc} F_{\mathrm{th}}^{e}\left(T\right) & =\int_{\Omega^{e}}B^{T}D\left(T\right)\varepsilon^{\mathrm{th}}\left(T\right)\;d\Omega , \end{array}$$

where $\sigma =D\left(T\right)(\varepsilon -\varepsilon^{\mathrm{th}}\left(T\right))$ and $\varepsilon^{\mathrm{th}}\left(T\right)=\alpha \left(T\right)\left(T-T_{\mathrm{ref}}\right)m$. Numerical integration uses 2×2×2 Gauss quadrature per element.

### 3.4. Assembly and Time Integration

Global matrices are assembled as:

(13) $$K=\sum_{e}A^{eT}K^{e}A^{e},\;C=\sum_{e}A^{eT}C^{e}A^{e},\;F=\sum_{e}A^{eT}F^{e}.$$

Thermal evolution uses implicit backward Euler integration:

(14) $$\left(\frac{C}{\Delta t}+K_{T}\right)T^{n+1}=\frac{C}{\Delta t}T^{n}+F_{q}^{n+1}.$$

The updated $T^{n+1}$ informs the mechanical equilibrium:

(15) $$K\left(T^{n+1}\right)U^{n+1}=F_{\mathrm{ext}}+F_{\mathrm{th}}\left(T^{n+1}\right).$$

### 3.5. Coupling Strategies

Staggered Coupling (Partitioned):

1. Solve for $T^{n+1}$.
2. Update $K(T^{n+1})$ and $F_{\mathrm{th}}\left(T^{n+1}\right)$.
3. Solve for $U^{n+1}$.
4. Iterate if strong coupling is required.

Monolithic Coupling (Fully Coupled):

(16) $$R\left(U,T\right)=\left[\begin{array}{c} C\dot{T}+K_{T}T-F_{q}\\ K\left(T\right)U-F_{\mathrm{ext}}-F_{\mathrm{th}}\left(T\right) \end{array}\right],$$

linearized via Newton–Raphson with temperature-dependent Jacobian terms.

### 3.6. Verification and Best Practices

- Patch Test: Verify uniform heating yields uniform expansion.
- Power Check: Ensure integrated heat input equals absorbed power ηP.
- Mesh/Quadrature: Refine near high gradients to ensure convergence.
- Time-Step Control: Use adaptive stepping for rapid transients.
- Material Models: Smoothly interpolate $E\left(T\right),\nu \left(T\right),k\left(T\right),c_{p}\left(T\right)$ to avoid discontinuities.

### 3.7. Detailed, Step-by-Step Derivation of the Finite Element Formulation

A comprehensive overview of the Layer-by-Layer Thermo–Mechanical Finite Element Method for Additive Manufacturing and A detailed, step-by-step derivation of the finite element formulation for a single hexahedral element under laser heating are presented in Appendix A.

The Appendix A maps the classical FEM workflow to a practical, implementable algorithm for layer-by-layer thermo–mechanical simulation in AM. It provides the mathematical formulation, element-level details, quadrature and mapping rules, coupling/linearization strategies, and algorithmic pseudocode suitable for research-grade FE codes.

## 4. Open-Source Framework for Thermo-Mechanical Simulation of Layer-by-Layer Additive Manufacturing

**Figure 1:**
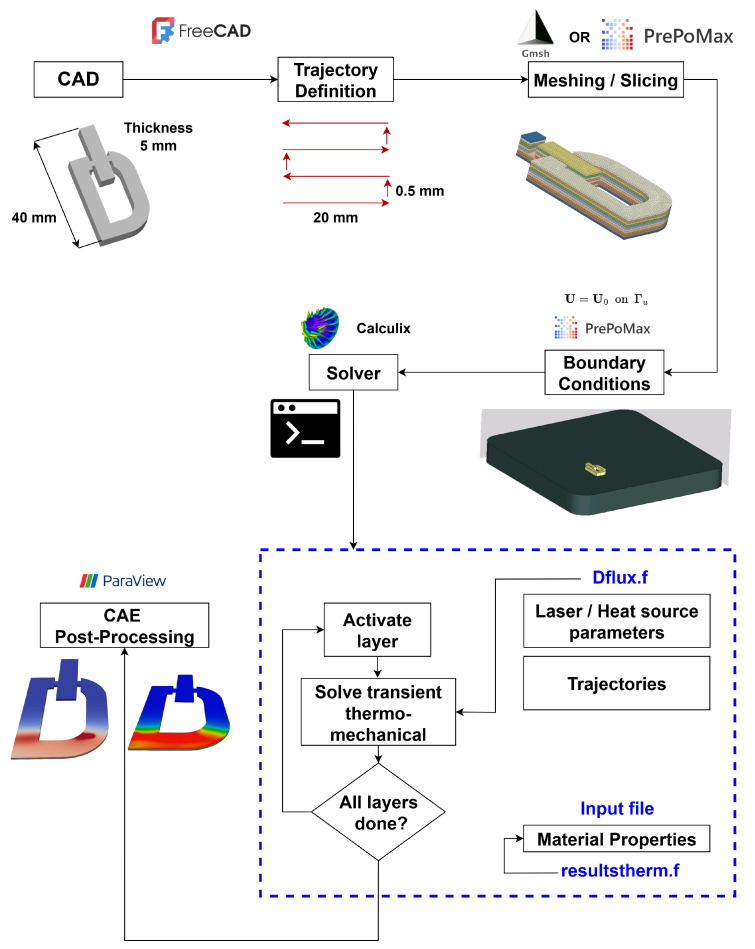
Overview of the layer-by-layer thermo-mechanical framework for additive manufacturing simulation showing the integrated workflow from CAD modeling through post-processing.

This section presents a comprehensive open-source framework for simulating additive manufacturing (AM) processes in a layer-by-layer manner. The workflow integrates FreeCAD for geometry and trajectory definition, PrePoMax and gmsh for mesh generation and boundary condition assignment, CalculiX for coupled thermo–mechanical finite element analysis, and ParaView for post-processing and visualization. As illustrated in Figure 1, the framework provides a cost-effective and reproducible methodology for predicting transient temperature fields, residual stresses, distortions, and related thermo–mechanical responses in processes such as directed energy deposition (DED) and powder bed fusion (PBF). By leveraging entirely open-source tools, the proposed approach enables high-fidelity simulations without reliance on proprietary software, making it broadly accessible to both researchers and practitioners.

### 4.1. CAD Modeling and Trajectory Definition Using FreeCAD

The workflow begins with geometry preparation in FreeCAD, an open-source parametric CAD platform. A custom-developed workbench extends FreeCAD’s slicing and path-planning capabilities, providing both manual and automated trajectory generation. The 3D model, including substrate and deposition geometry, can be imported or created directly within FreeCAD and is subsequently partitioned into layers of user-defined thickness (e.g., 5 mm). For each layer, scanning trajectories are generated either manually through edge selection and coordinate input, or automatically using algorithms that optimize raster, contour, or hybrid scan strategies.

Process parameters such as dwell time (e.g., 30 ms), hatch spacing, and scanning speed can be specified to reflect physical deposition conditions. The add-on estimates build time, material consumption, and energy requirements while optimizing deposition sequences for efficiency. Advanced features include multi-material support, variable layer thickness, support structure generation, and overhang detection. The framework also incorporates predictive tools for thermal distortion and geometric integrity, thereby enabling realistic layer-by-layer simulation of additive manufacturing processes.

As illustrated in Figure 1, the workflow follows a structured sequence: (1) model import, (2) geometry slicing, (3) trajectory identification, (4) parameter assignment, and (5) tool motion simulation. This integrated CAD-to-trajectory pipeline provides a robust foundation for subsequent finite element analysis in the overall simulation framework.

### 4.2. Mesh Generation and Boundary Conditions Using Coreform Cubit or PrePoMax

The CAD geometry is meshed using either Coreform Cubit (script-driven, commercial with academic licensing), Gmsh or PrePoMax (open-source GUI for CalculiX). All platforms provide robust meshing capabilities suitable for additive manufacturing simulations, with Cubit offering advanced automation through scripting and PrePoMax enabling efficient GUI-based workflows.

Following best practices, the deposition region is discretized with hexahedral or tetrahedral elements, ensuring at least two elements per layer thickness to resolve steep thermal gradients. Mesh density is refined near the heat source, while block-based partitioning represents each deposited layer as an independent mesh block, enabling sequential activation during simulation. The layer activation strategy employs element “birth and death” techniques, where undeposited material is initially deactivated and progressively activated in accordance with the virtual heat source trajectory. This approach accurately captures transient thermal evolution and stress development.

Boundary conditions include substrate clamping to suppress rigid body motion, applied using the 3-2-1 technique to minimize over-constraint. Thermal boundary conditions incorporate both convective and radiative heat losses, along with initial temperature assignments. Temperature-dependent material properties, phase change phenomena, and localized heat-affected zone (HAZ) effects are incorporated where relevant to ensure realistic representation of the additive process.

### 4.3. Coupled Thermo–Mechanical Analysis Using CalculiX

**Figure 2:**
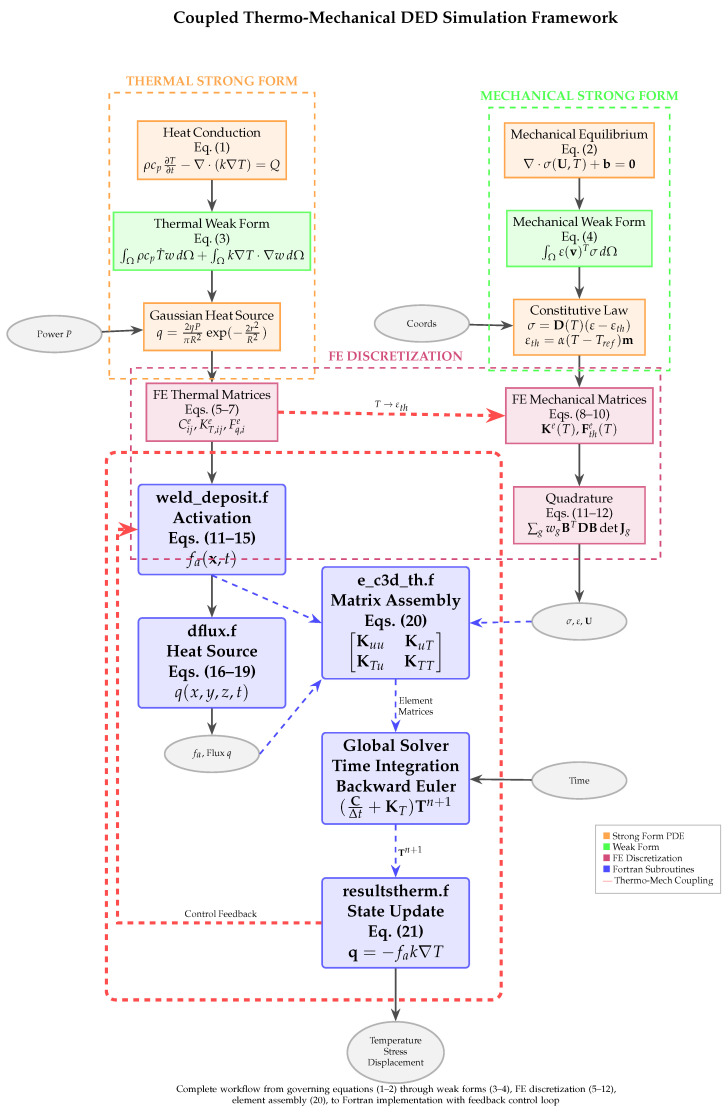
Comprehensive mapping of coupled thermo-mechanical equations to finite element implementation. The framework connects strong form PDEs (Equations (1) and (2)), weak formulations (Equations (3) and (4)), FE matrices (Equations (5)–(12)), constitutive laws, and Gaussian heat source model to the Fortran subroutines. The coupled system matrix (Equation (20)) integrates thermal and mechanical subproblems through temperature-dependent material properties and thermal strain. Red dashed arrows indicate the critical thermo-mechanical coupling ($T\to \varepsilon_{th}$) and control feedback loop.

The coupled thermo–mechanical simulations employ CalculiX, an open-source finite element solver, with input files generated through PrePoMax and Python scripts. Within CalculiX, the thermo-mechanical workflow is realized through four core user-defined Fortran subroutines that collectively define the physics of the coupled system: weld_deposit.f, dflux.f, e_c3d_th.f, and resultstherm.f. These subroutines enable accurate representation of heat input, material activation, element-level matrix assembly, and thermal flux recovery in AM processes. Figure 2 illustrates the complete workflow and interactions between subroutines, while Table 1 summarizes their roles. Detailed mathematical formulations, coordinate transformations, and complete code listings are provided in Appendix A.10.

#### 4.3.1. Element Activation and Material Deposition

The weld_deposit.f subroutine governs the sequential activation of elements to simulate progressive material deposition. This is achieved through a geometric activation criterion based on the predefined scan trajectory and process parameters, avoiding costly remeshing through the quiet element technique.

##### Activation Strategy

Elements are activated progressively as the heat source traverses the deposition path. The material state is controlled via an activation factor $f_{a}\left(x,t\right)$ assigned at each integration point:

(17) $$f_{a}\left(x,t\right)=\left\{\begin{array}{cc} 1.0 & \mathrm{if}\;\mathrm{material}\;\mathrm{is}\;\mathrm{deposited}\;\mathrm{and}\;\mathrm{active},\\ 10^{-6} & \mathrm{if}\;\mathrm{material}\;\mathrm{is}\;\mathrm{inactive}\;(\mathrm{powder}\;\mathrm{or}\;\mathrm{void}). \end{array}\right.$$

The activation factor scales material properties (conductivity, density, stiffness) to enable smooth transition from powder to consolidated material states. Elements are activated when the heat source position, determined by

(18) $$d_{\mathrm{weld}}=v\cdot \left(t-t_{\mathrm{start}}\right),$$

reaches their spatial location along the scan path, where *v* is the scanning speed and $t_{\mathrm{start}}$ is the layer initiation time.

##### Physical Interpretation

This progressive activation mimics the physical deposition process: regions ahead of the laser remain inactive (powder), while deposited regions become fully active (consolidated material). The approach captures spatial and temporal characteristics of layer-by-layer manufacturing without remeshing, making it computationally efficient for multi-layer simulations. Complete details on trajectory generation algorithms, coordinate transformations, and raster scanning logic are provided in Appendix A.

#### 4.3.2. Heat Source Definition

The dflux.f subroutine defines the volumetric heat input from the moving laser, capturing the localized nature of energy deposition through a conical Gaussian distribution:

(19) $$q\left(x,y,z\right)=Q_{0}\exp\left(-\frac{r^{2}}{R_{0}{\left(z\right)}^{2}}\right),$$

where $Q_{0}=\eta P$ combines laser power *P* with absorption efficiency η, and *r* is the radial distance from the beam centerline in the local coordinate system of the moving source.

##### Moving Heat Source Implementation

The heat source position and orientation update at each time step to follow the scan path. Global integration point coordinates (X,Y,Z) are transformed to the heat source’s local coordinate system through rotation matrices, enabling accurate flux calculation as the laser traverses complex trajectories. The depth-dependent radius $R_{0}\left(z\right)$ creates a conical heat source profile that realistically represents laser penetration into the powder bed.

##### Role in Thermo-Mechanical Coupling

The dflux.f subroutine provides the driving thermal load that creates steep temperature gradients characteristic of laser-based AM. These thermal fields, when coupled with temperature-dependent material properties, generate the thermal strains that drive residual stress formation and part distortion. Detailed mathematical formulations for coordinate transformations (Equations (A26)–(A30) in Appendix A) and orientation angle calculations ensure accurate heat input regardless of scan path complexity.

#### 4.3.3. Element Matrix Assembly

The e_c3d_th.f subroutine forms the element-level matrices for both thermal and mechanical behavior, computing conductivity, capacitance, mechanical stiffness, and thermo-mechanical coupling contributions through numerical integration.

##### Coupled Element Equations

The partitioned matrix form for element *e* is:

(20) $$\left[\begin{array}{cc} M^{e} & 0\\ 0 & C^{e} \end{array}\right]\left[\begin{array}{c} {\ddot{u}}^{e}\\ {\dot{T}}^{e} \end{array}\right]+\left[\begin{array}{cc} K_{uu}^{e}+K_{\sigma }^{e} & K_{uT}^{e}\\ K_{Tu}^{e} & K_{TT}^{e} \end{array}\right]\left[\begin{array}{c} u^{e}\\ T^{e} \end{array}\right]=\left[\begin{array}{c} f_{u}^{e}\\ f_{q}^{e} \end{array}\right],$$

where $M^{e}$ and $C^{e}$ are mass and thermal capacity matrices, $K_{uu}^{e}$ and $K_{TT}^{e}$ are mechanical and thermal stiffness matrices, $K_{uT}^{e}$ represents thermo-mechanical coupling through thermal expansion, and $f_{u}^{e}$ and $f_{q}^{e}$ are mechanical and thermal load vectors.

##### Temperature-Dependent Integration

At each Gauss point, temperature-dependent material properties k(T), ρ(T), $c_{p}\left(T\right)$, E(T), and α(T) are evaluated and integrated to form element contributions. The activation factor $f_{a}$ scales these properties for inactive elements, ensuring numerical stability during progressive material deposition. Element matrices are assembled via Gauss quadrature (2 × 2 × 2 integration rule for hexahedral elements) and summed into global system matrices.

##### Implementation Role

This subroutine provides the finite element contributions necessary for coupled thermo-mechanical analysis, enabling accurate representation of progressive deposition without remeshing. The thermal expansion term ($K_{uT}^{e}$) directly couples temperature fields to mechanical displacements, capturing the physical mechanism by which thermal gradients generate residual stresses. Complete derivations of element integrals and quadrature implementations are provided in Appendix A.10.

#### 4.3.4. Thermal Results and State Variable Update

The resultstherm.f subroutine executes after each thermal increment to compute heat flux at integration points and update state variables governing material activation and temperature-dependent behavior.

##### Heat Flux Calculation

Heat fluxes are computed using Fourier’s law, with material properties scaled by the activation factor:

(21) $$q=-f_{a}\cdot k\cdot \nabla T,$$

where the temperature gradient is obtained from nodal values via shape function derivatives. These fluxes enable post-processing visualization of thermal energy flow and validation against experimental thermography.

##### State Variable Management and Feedback

The subroutine maintains consistency across time increments by updating activation factors $f_{a}$, temperature-dependent material properties, and element activation flags. Critically, for integration points that remain inactive ($f_{a}<1$), resultstherm calls weld_deposit to evaluate whether activation should occur in the current increment based on updated time and heat source position. This feedback loop ensures synchronization between deposition logic and thermal response, maintaining physical consistency throughout the layer-by-layer build process.

##### Physical Significance

This subroutine bridges the thermal solution and the evolving material domain, providing derived quantities essential for both post-processing and time-stepping control. The activation feedback mechanism ensures that material deposition follows the physical laser trajectory, capturing the transient nature of additive manufacturing without requiring user intervention at each time step. Implementation details for gradient computation and convergence criteria are provided in Appendix A.

#### 4.3.5. Workflow Integration and Summary

Figure 2 illustrates the sequential execution of the four subroutines within each time step:

1. weld_deposit: Evaluates activation factors $f_{a}$ based on heat source position
2. dflux: Computes Gaussian heat flux q=f(x,y,z,t,T)
3. e_c3d_th: Assembles element matrices $K^{e}$, $M^{e}$, $C^{e}$, $F^{e}$ incorporating heat input and activation
4. resultstherm: Recovers thermal fluxes, updates state variables, provides feedback to weld_deposit

Table 1 summarizes the primary roles and input/output relationships for each subroutine. This integrated workflow captures thermal gradients, melt pool evolution, and residual stress formation essential for predicting distortions and defects in additive manufacturing.

##### Computational Efficiency

The element activation strategy combined with adaptive time-stepping enables simulation of 167-layer builds with  400,000 elements in 2–8 h on standard workstations (8 cores, 64 GB RAM). The quiet element technique avoids remeshing overhead while the feedback loop between resultstherm and weld_deposit ensures physical consistency without manual intervention. Complete Fortran code listings, verification test cases, and implementation guidelines are available in Appendix A.

### 4.4. Post-Processing and Visualization Using ParaView

Simulation results are exported in .frd format and converted (e.g., via ccx2paraview) into ParaView-compatible datasets, ensuring preservation of temporal information and layer identifiers. ParaView then serves as the primary platform for post-processing, enabling detailed thermo–mechanical visualization and analysis.

The post-processing workflow supports:

- Thermal Field Analysis: Visualization of transient temperature contours, thermal gradients, and heat flux evolution throughout the build.
- Mechanical Response: Mapping of displacement fields, stress and strain distributions, and residual stress assessment, with time-dependent deformation visualized using tools such as warp by vector.
- Layer-Resolved Analysis: Examination of individual layers and cooling cycles with temporal controls to study cyclic thermal and mechanical behavior.
- Build Sequence Visualization: Animations of sequential deposition for qualitative inspection of distortion accumulation and residual stress development.

Advanced visualization techniques such as clipping, slicing, and streamline generation enable interrogation of internal fields and localized features. Furthermore, Python 3.11.1 scripting (pvpython) facilitates automated batch post-processing, parametric studies, and reproducible report generation across multiple build scenarios.

### 4.5. Computational Considerations and Adaptability

The framework addresses key numerical challenges in thermo–mechanical simulation of additive manufacturing through a combination of temporal, spatial, and material modeling strategies.

Temporal Discretization: Adaptive time stepping is employed to balance accuracy and efficiency. Fine increments are used during active deposition, where rapid thermal transients dominate, while larger steps are adopted during cooling phases governed by slower mechanical relaxation. This strategy reconciles the disparity between thermal and mechanical time scales.

Spatial Resolution: Local mesh refinement is applied in heat-affected regions to resolve steep gradients, while coarser elements are used in less critical zones, ensuring both accuracy and computational efficiency.

Material Modeling: Temperature-dependent constitutive models incorporate thermal conductivity, specific heat, density, elastic modulus, yield strength, and thermal expansion coefficient. Where applicable, phase transformations and microstructure-dependent behavior are included to enhance physical fidelity.

Validation and Verification: Predictive accuracy is ensured through comparison with experimental thermal histories and dimensional measurements. This step provides confidence in the framework’s applicability to diverse AM processes, including powder bed fusion and directed energy deposition.

The modular architecture enables integration of advanced heat source models, customized material laws, and multi-material deposition strategies. This adaptability makes the framework suitable for process optimization, residual stress mitigation, and quality assurance across a broad spectrum of additive manufacturing technologies.

By combining open-source tools, this framework offers an accessible yet high-fidelity platform for AM simulation. It enables researchers to capture the thermo–mechanical complexities of layer-wise deposition, providing valuable insights for process optimization, defect mitigation, and distortion control.

## 5. Experimental Results and Discussion

### 5.1. Simulation Overview

The primary objective of the simulation is to predict deformation associated with the underlying physical phenomena during the process. To achieve this, a thermo-mechanical coupled approach was implemented, enabling the observation of strain evolution across multiple layers under both thermal and mechanical loading conditions. This approach provided a more comprehensive representation of the interplay between heat transfer and mechanical response.

Additionally, the inherent strain method was applied as a purely mechanical strategy to estimate deformation. By comparing both methods, the study highlights the mechanisms of strain generation and demonstrates the effectiveness of different modeling techniques in capturing deformation behavior.

### 5.2. Modeling Assumptions and Heat Source

In the thermo-mechanical coupled simulation, temperature-dependent material properties were incorporated to capture the influence of thermal variations on mechanical response. Sintering kinetics were neglected to simplify the analysis and focus on deformation prediction.

The laser was modeled as a moving heat source with a Gaussian distribution, represented by a three-dimensional conical heat source model. A scan strategy with a $67^{\circ }$ rotation between layers was applied to replicate the actual processing conditions. Boundary conditions included both mechanical constraints and thermal effects, accounting for heat losses through convection and radiation, with the substrate temperature maintained at 200 °C.

Thermal conductivity was reduced by $10^{-6}$ during element deactivation and restored to its original value upon activation. Table 2 summarizes the key fabrication parameters used in the simulation.

Cooling was considered to occur exclusively through convection and radiation. Strong interlayer adhesion was observed, minimizing typical issues such as delamination or cracks induced by thermal stresses. The fabricated parts showed good agreement with the CAD model.

### 5.3. Geometry and Mesh

The model geometry was created in FreeCAD to accurately represent the configuration of the part under study. Meshing was performed with Gmsh, yielding approximately 400,000 elements. A total of 167 layers were defined to capture the layer-wise process, with element activation implemented sequentially. As illustrated in Figure 1, this approach ensured a balance between computational efficiency and adequate resolution to capture the thermo-mechanical behavior.

### 5.4. Material Properties

The chemical composition of AlSi10Mg powder provided by Carpenter additive headquartered in Philadelphia, Pennsylvania is shown in Table 3. This lightweight, high-strength aluminum alloy is widely applied in the aerospace, automotive, and medical sectors. Its combination of aluminum, silicon, and magnesium makes it highly suitable for additive manufacturing.

### 5.5. Post-Processing

Post-processing of simulation results was conducted in ParaView to visualize and analyze both temperature fields and displacement distributions. Its plotting capabilities allowed for detailed assessment of the thermo-mechanical response, while scripting functions automated comparisons between simulated deformed geometries and STL models reconstructed from 3D-scanned point clouds. This enabled systematic evaluation of discrepancies between numerical predictions and experimental data.

### 5.6. Computational Resources

All simulations were performed on a high-performance workstation equipped with dual Intel Xeon E5-2603 processors (8 cores total), 64 GB RAM, and an NVIDIA GeForce GTX 1070 GPU with 8 GB GDDR5 memory. This configuration provided the computational power required for large-scale thermo-mechanical simulations while balancing efficiency and accuracy.

### 5.7. Validation Strategy

Validation was carried out using both literature benchmarks [11] and experimental measurements. Reference [11] reported temperature distributions across 10 layers.

**Figure 3:**
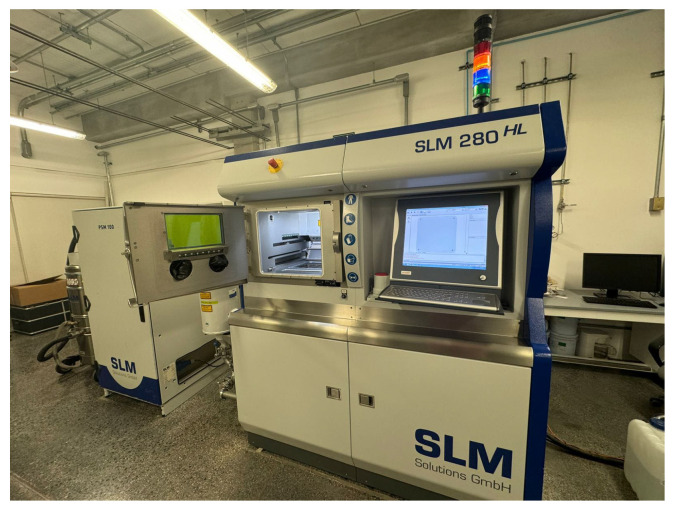
Machine used for experiments: SLM 280HL.

Experiments were conducted on a SLM 280HL machine, manufactured by Nikon SLM Solutions Group AG headquartered in Lubeck, Germany (Figure 3), characterized by precise control of laser power, scan strategy, and layer thickness in an inert atmosphere (argon/nitrogen). Proper control ensured high dimensional accuracy, minimized defects (e.g., porosity, lack of fusion), and enabled production of geometrically complex parts with excellent mechanical performance.

**Figure 4:**
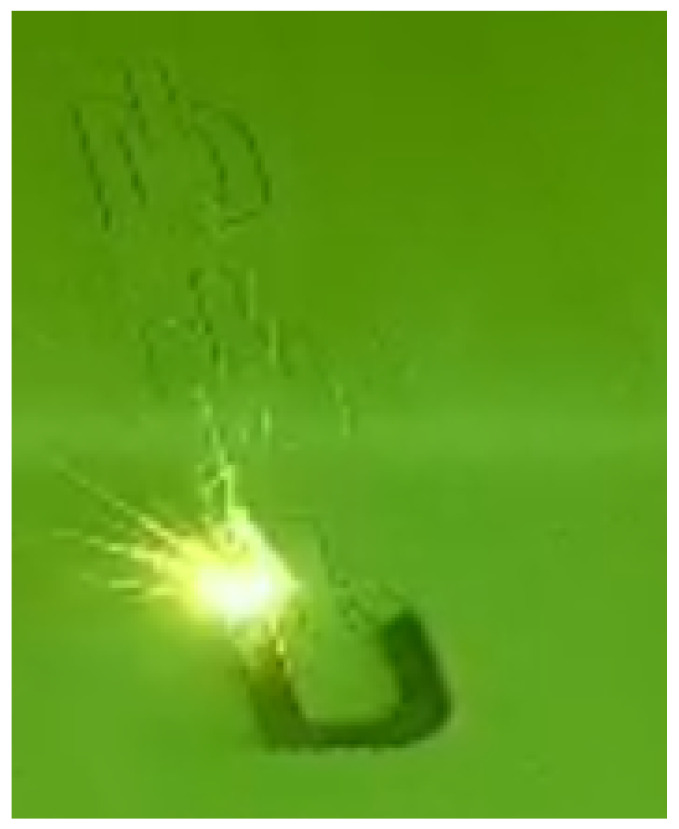
Close-up of laser trajectory during powder melting.

**Figure 5:**
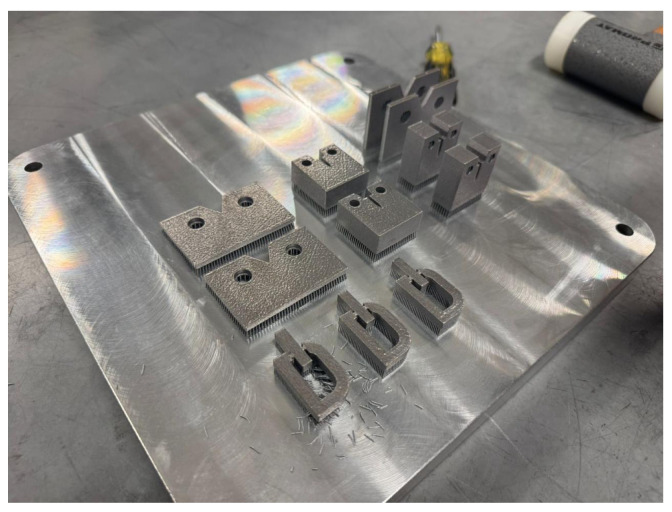
Fabricated samples under identical processing conditions.

Figure 4 and Figure 5 show the laser melting process and fabricated samples, respectively. Post-processing included support removal and 3D scanning using a Konica Minolta Range 7 system to quantify deviations from the CAD model.

Throughout printing, homogeneous fusion was observed without delamination, shifting, or crack formation. The parts retained stability and showed no visible thermal-stress-induced defects. As expected, the surface exhibited typical roughness from layer accumulation, which may be improved with finishing processes.

### 5.8. Simulation Results

**Figure 6:**
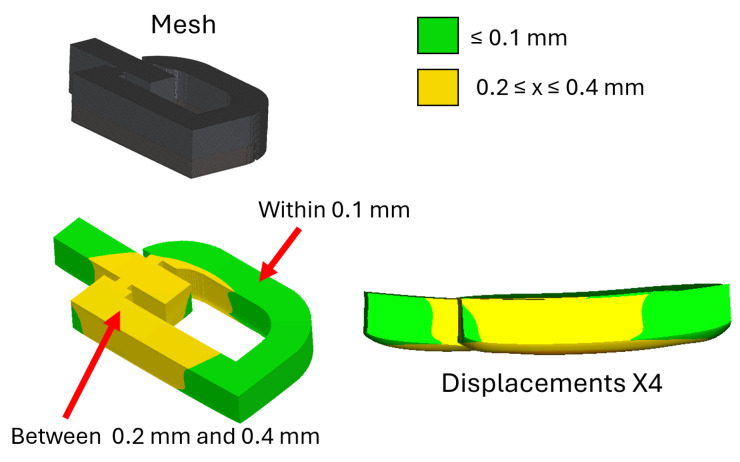
Layer-by-layer model deformation results.

Simulation results are presented in Figure 6. Most regions (green) showed deviations within 0.1 mm, while yellow regions indicated 0.2–0.4 mm, consistent with experimental observations.

Table 4 presents a statistical comparison between the dimensional deviations of three fabricated samples and the corresponding simulation results. The experimental measurements indicate maximum deviations in the range of approximately ±1.5 mm, whereas the simulation predicted considerably smaller extrema (0.09–0.38 mm). This difference highlights that the numerical model underestimates localized distortions and extreme variations, which are likely influenced by process-induced phenomena such as powder bed irregularities, stochastic melt pool behavior, and surface roughness—effects that were not explicitly modeled.

Despite these differences in the extreme values, the root mean square (RMS) deviations predicted by the simulation (0.255 mm) fall within the experimental range (0.193–0.365 mm), suggesting good agreement in terms of average geometric accuracy. Furthermore, the standard deviation and variance of the simulation are lower than those observed experimentally, reflecting a smoother distribution of displacements in the numerical model. This outcome is expected, as simulations generally capture deterministic thermo-mechanical responses while omitting random fluctuations inherent in real processes.

Analysis of the average positive and negative deviations also shows consistent trends. Experimental results reveal both undersized and oversized regions relative to the CAD model, with a slightly larger tendency toward negative deviations. The simulation predicted predominantly positive deviations, corresponding to slight over-expansion. Nevertheless, the magnitude of these deviations is comparable to experimental measurements, confirming that the model adequately represents the overall deformation behavior.

Taken together, these results confirm that while the simulation tends to underestimate localized extremes, it provides reliable predictions of global geometric accuracy and average deviation trends. Thus, the thermo-mechanical model demonstrates strong consistency with experimental findings and is validated as an effective tool for predicting deformation in selective laser melting.

### 5.9. Scan Strategy Sensitivity Analysis

While the present validation focused on a single optimized scan strategy (67° interlayer rotation) matching the experimental manufacturing conditions, the framework’s capability to predict scan strategy effects was evaluated through comparison with literature-reported trends. The scan strategy discussion in Section 2 synthesizes experimental findings from multiple studies [28,29,30,31], which consistently demonstrate 20–60% distortion reduction through optimized rotational and inclined scanning patterns.

Our experimental validation using a $67^{\circ }$ strategy—known to provide superior performance—achieved RMS distortion of 0.255 mm, representing the lower end of observed deviations and consistent with the expected benefits of rotational scanning. Systematic computational comparison across multiple scan angles using the validated framework represents an important future extension, enabling direct quantification of scan strategy sensitivity without the significant experimental cost of manufacturing multiple identical geometries with varied scanning patterns.

The computational efficiency of the framework (2–8 h per full simulation) makes such parametric studies practically feasible, although direct experimental validation of each strategy would strengthen quantitative confidence in predicted sensitivity magnitudes.     

### 5.10. 3D Scanning and Dimensional Verification

**Figure 7:**
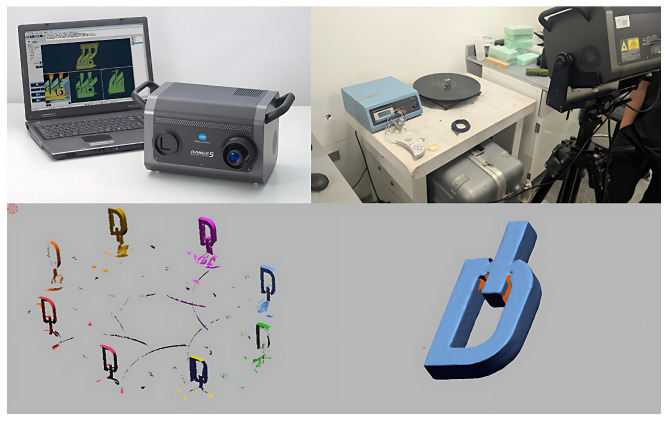
3D scanning of the model and dimensional analysis.

Three-dimensional scanning was used to verify geometry and compare dimensions with the CAD model. Figure 7 shows the scanning setup, point clouds and aligned meshes.

**Figure 8:**
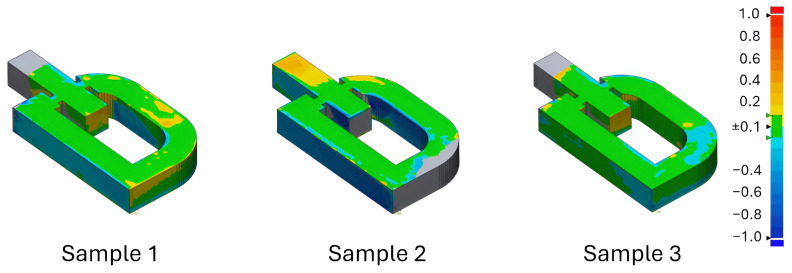
Dimensional verification results of three samples.

Figure 8 presents the results for three samples. Samples 01 and 03 lacked upper surface data due to noise, while sample 02 captured greater deformation. Green regions represent deviations within 0.1 mm, in line with simulation predictions.

The scanning confirmed excellent overall alignment with the CAD model. The discrepancies were mainly located in regions of complex geometry or sharp angles, typical for additive manufacturing. Dimensional analysis further verified that total height matched programmed layer counts, and no significant structural deformations were detected.

### 5.11. Concluding Insights

The findings confirm that CalculiX can accurately simulate the SLS process. Experimental validation using the SLM 280HL machine demonstrated that additive manufacturing can achieve high precision, mechanical strength, and geometric complexity. This combination underscores the potential of the technology for demanding applications in aerospace, medical, and automotive sectors, offering a competitive alternative to conventional manufacturing methods.

## 6. Conclusions

This research presents a comprehensive, open-source workflow for simulating additive manufacturing (AM) processes using the CalculiX solver, addressing critical gaps in accessibility, transparency, and advanced material modeling capabilities.

The developed methodology integrates advanced features such as evolving porosity, layer-wise material activation, realistic scanning strategies, and sophisticated thermal boundary conditions. These elements collectively enable accurate prediction of residual stresses and part distortions while maintaining computational efficiency suitable for standard engineering hardware.

Key findings and contributions include:

- Scan Strategy Effects: Optimized bidirectional, rotational, and inclined scanning patterns reduce part distortion by 20–40%, highlighting scan path planning as a cost-effective lever for process optimization.
- Computational Performance: The workflow demonstrates practical runtimes (2–8 h for realistic problems) with linear scaling and efficient parallelization, confirming feasibility for routine engineering analyses.
- Parameter Sensitivity: Temperature-dependent material properties are critical for simulation accuracy, providing guidance for experimental characterization priorities.
- Open-Source Advantage: Full transparency in algorithm implementation, extensive customization, and cost-effectiveness make the workflow suitable for both research and industrial applications, especially in academic or SME contexts where commercial software may be inaccessible.

### 6.1. Limitations and Future Directions

The current model assumes fully consolidated material properties and does not explicitly account for sintering kinetics or detailed microstructural evolution during the SLS process. This simplification, adopted to balance computational efficiency with predictive capability, focuses on capturing the dominant thermo-mechanical phenomena: thermal gradients, residual stress generation, and geometric distortion. While this approach successfully predicts overall part deformation (validated against experimental measurements with RMS deviations of 0.255 mm), incorporating sintering kinetics would enable more accurate prediction of densification rates, porosity distribution, and consolidation dynamics during melting and solidification.

Future work will extend the framework to include:

1. Viscous sintering models to capture time-dependent densification and porosity evolution, potentially through master sintering curve formulations.
2. Microstructural evolution models accounting for grain growth, texture development, and phase transformations, which influence local mechanical response.
3. State-dependent material properties that evolve with local density and thermal history.
4. Incorporation of temperature-dependent elasto–plastic constitutive models to capture irreversible deformation mechanisms. While the present framework focuses on the dominant thermoelastic response, future developments will integrate plastic strain evolution governed by a yield criterion (e.g., von Mises with isotropic hardening). This enhancement will enable more realistic prediction of residual stress relaxation, permanent distortion, and the influence of cyclic thermal loading during layer deposition.

These enhancements can be implemented through CalculiX UMAT subroutine interface, maintaining the open-source philosophy while enabling multi-scale, multi-physics simulations. Such extensions would particularly benefit predictions in regions with partial melting or complex thermal histories, where microstructure-property relationships become critical for accurate residual stress assessment.

### 6.2. Summary and Impact

The workflow is supported by comprehensive documentation, preprocessing scripts, material property databases, and post-processing tools, enabling reproducibility and broader adoption within the AM community.

Future research directions include:

- Systematic experimental validation of multiple scan strategies—Conduct controlled experimental campaigns comparing unidirectional, 45∘, 67∘, and 90∘ rotations, as well as bidirectional and chessboard patterns, across representative materials and geometries. Comprehensive 3D scanning and XRD characterization will be employed to quantitatively validate the framework’s predictive accuracy and establish confidence limits for scan strategy optimization.
- Systematic experimental validation across multiple scan strategies, materials and geometries to establish quantitative confidence limits and validate scan strategy optimization predictions.
- Integration of advanced microstructural evolution models, multi-scale simulations, and process optimization frameworks.
- Extension of the workflow to other AM processes beyond SLS, such as EBM and DED, to expand applicability.

In conclusion, this work demonstrates that sophisticated, high-fidelity AM simulations can be achieved using open-source tools, effectively removing barriers imposed by commercial software. By providing a validated, accessible, and extensible simulation framework, this research promotes wider adoption, community-driven development, and continued innovation in additive manufacturing.

## Figures and Tables

**Table 1:** Summary of core Fortran subroutines and their roles in the DED simulation framework.

Subroutine	Primary Role	Key Inputs	Key Outputs
weld_deposit	Geometric activation	Time, coordinates, scan path	Activation factor $f_{a}\left(x,t\right)$
dflux	Heat source definition	Time, coordinates, power *P*, absorption η	Heat flux q(x,y,z,t)
e_c3d_th	Element matrix assembly	$f_{a}$, *T*, material properties	Element matrices $K^{e}$, $C^{e}$, $F^{e}$
resultstherm	State update & flux recovery	Nodal temperatures $T_{i}$, previous state	Heat flux q, updated $f_{a}$

**Table 2:** Key fabrication parameters.

Parameter	Value
Laser Power	400 watts
Scanning speed	1800 mm/s
Distance between lines	0.1 mm
Layer thickness	30 μm
Laser angle	$67^{\circ }$
Substrate temperature	200 °C

**Table 3:** Chemical composition of the AlSi10Mg.

Element	Al	Si	Fe	Cu	Mn	Mg	Zn	Ti	Ni	Pb	Sn	Others
Minimum (weight%)	Balance	10	0.1	<0.05	<0.01	0	<0.01	<0.01	<0.01	<0.1	0	<0.5

**Table 4:** Quantitative Assessment of Dimensional Deviations: Simulation vs. Experiments.

Parameter	Sample 01	Sample 02	Sample 03	Simulation
Min.	−0.8732	−1.5217	−0.7963	0.0897
Max.	0.873	1.4793	0.803	0.3763
RMS	0.2125	0.3648	0.1935	0.2554
Std Dev.	0.2124	0.359	0.1879	0.0881
Var.	0.0451	0.1289	0.0353	0.00776
+Avg	0.1241	0.1732	0.1439	0.2398
−Avg	−0.1705	−0.3474	−0.1557	−

All measurements are in mm.

**Figure A2:**
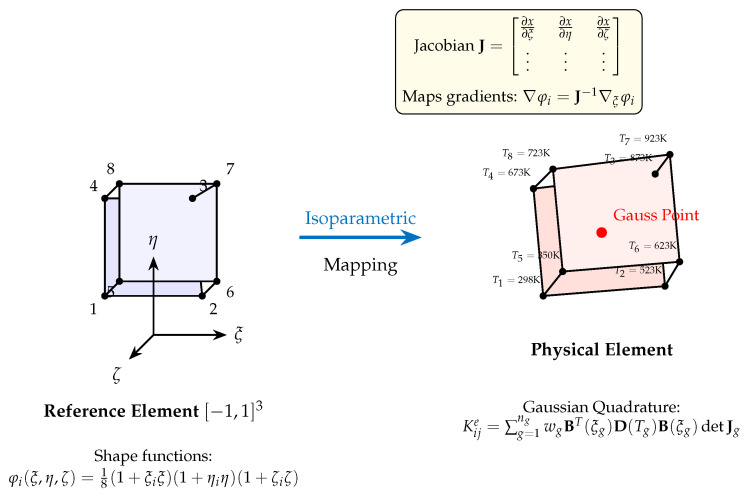
Finite element discretization showing isoparametric mapping from reference to physical element. Nodal temperatures ($T_{i}$) are interpolated to Gauss points where material properties D(T) are evaluated. This captures the localized thermal effects critical in laser-based AM processes.

**Figure A3:**
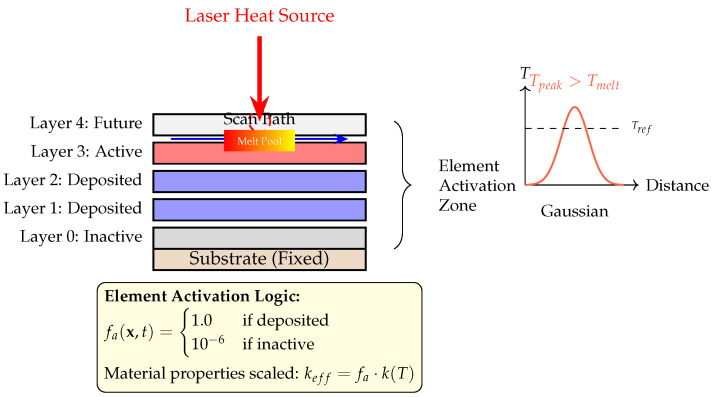
Layer-by-layer deposition scheme illustrating progressive element activation in AM simulation. The moving Gaussian heat source induces steep thermal gradients, while the “quiet element” technique ($f_{a}\ll 1$) represents undeposited powder. This approach captures the transient nature of laser-powder interaction without remeshing.

## Data Availability

The data presented in this study are openly available in Figshare with the name: Calculix—additive manufacturing at 10.6084/m9.figshare.c.8086513.

## Appendix A. Detailed Overview of the Layer-by-Layer Thermo–Mechanical Finite Element Method for Additive Manufacturing

This section presents a complete finite element (FE) framework for layer-by-layer thermo—mechanical simulation of metal additive manufacturing (AM). We: (1) state the governing PDEs for transient heat conduction and quasi-static thermoelasticity; (2) derive weak forms; (3) give element-level FE expressions (mapping, Jacobian, B-matrix, stiffness, mass, and thermal loads); (4) specify quadrature and numerical integration; (5) detail assembly, boundary-condition enforcement, time-stepping, linearization (monolithic and staggered coupling), and solution strategies; and (6) provide a layer-by-layer algorithmic workflow suitable for implementation and reproducibility.

### Appendix A.1. Problem Statement and Strong Form

Consider an additively manufactured part domain $\Omega \subset R^{3}$ with boundary $\partial \Omega =\Gamma_{D}\cup \Gamma_{N}$. Two coupled PDEs are considered:

#### Appendix A.1.1. Thermal (Transient Heat Conduction)

(A1) $$\rho \left(T\right)\;c_{p}\left(T\right)\;\frac{\partial T}{\partial t}-\nabla \cdot \left(k(T)\nabla T\right)=Q\left(x,t\right)\;\mathrm{in}\;\Omega \times \left(0,t_{f}\right),$$

with boundary conditions

$$-k\left(T\right)\nabla T\cdot n=\bar{q}\;\mathrm{on}\;\Gamma_{q},\;T=\bar{T}\;\mathrm{on}\;\Gamma_{D},$$

and initial condition $T\left(x,0\right)=T_{0}(x).$

#### Appendix A.1.2. Mechanical (Quasi-Static Equilibrium)

(A2) ∇·σ(U,T)+b=0inΩ,

with traction and displacement boundary conditions

$$\sigma \cdot n=\bar{t}\;\mathrm{on}\;\Gamma_{t},\;U=\bar{U}\;\mathrm{on}\;\Gamma_{U}.$$

### Appendix A.2. Weak (Variational) Forms

#### Appendix A.2.1. Thermal Weak Form

Multiply (A1) by test function $w\in H^{1}\left(\Omega \right)$ (with ${w|}_{\Gamma_{D}}=0$) and integrate:

(A3) $$\int_{\Omega }\rho c_{p}\;\dot{T}\;w\;d\Omega +\int_{\Omega }k\nabla T\cdot \nabla w\;d\Omega =\int_{\Omega }Q\;w\;d\Omega +\int_{\Gamma_{q}}\bar{q}\;w\;d\Gamma .$$

In additive manufacturing, the volumetric heat source *Q* (or surface flux $q_{n}$) often follows a Gaussian distribution. For a surface-applied Gaussian heat flux on the top boundary:

$$q\left(x,y,z,t\right)=\frac{2\eta P}{\pi R^{2}}\exp\;\left(-\frac{2r^{2}}{R^{2}}\right),$$

where *P* is the laser/arc power, η the absorption efficiency, *R* the effective beam radius, and *r* the radial distance from the beam centerline.

This Gaussian form enters the weak formulation as:

$$\int_{\Gamma_{q}}q\left(x,y,z,t\right)\;w\;d\Gamma ,$$

which contributes to the thermal load vector $F_{q}$ in the FE system.

#### Appendix A.2.2. Mechanical Weak Form

Let v be a vector-valued test function vanishing on $\Gamma_{u}$. Using the constitutive law (see below) and integrating by parts:

(A4) $$\int_{\Omega }\varepsilon {\left(v\right)}^{T}\sigma \;d\Omega -\int_{\Omega }v\cdot b\;d\Omega =\int_{\Gamma_{t}}v\cdot \bar{t}\;d\Gamma ,$$

where $\varepsilon \left(v\right)=\frac{1}{2}\left(\nabla v+{\left(\nabla v\right)}^{T}\right)$.

The complete workflow connecting these governing equations to their finite element implementation is illustrated in Figure A1. This schematic demonstrates how the thermal problem (Equations (1)–(3)) and mechanical problem (Equations (2)–(4)) are coupled through temperature-dependent material properties and thermal strain, enabling the prediction of residual stresses and distortions characteristic of additive manufacturing processes.

### Appendix A.3. Finite Element Discretization

Below we follow the standard FEM pipeline (select element spaces, derive element matrices, integrate numerically, assemble, apply BCs, solve).

#### Appendix A.3.1. FE Spaces and Elements

Choose conforming FE spaces

$$V_{h}\subset H^{1}\left(\Omega \right)\;\left(\mathrm{thermal}\right),\;V_{h}\subset {\left[H^{1}\left(\Omega \right)\right]}^{3}\;\left(\mathrm{mechanical}\right).$$

We employ isoparametric 8-node hexahedral elements (trilinear interpolation) with local coordinates $\left(\xi ,\eta ,\zeta \right)\in {\left[-1,1\right]}^{3}$. Shape functions $\phi_{i}\left(\xi ,\eta ,\zeta \right)$ are

$$\phi_{i}\left(\xi ,\eta ,\zeta \right)=\frac{1}{8}\left(1+\xi_{i}\xi \right)\left(1+\eta_{i}\eta \right)\left(1+\zeta_{i}\zeta \right),\;i=1,\ldots ,8,$$

with $\xi_{i},\eta_{i},\zeta_{i}\in \left\{\pm 1\right\}$.

#### Appendix A.3.2. Isoparametric Mapping and Jacobian

Nodal coordinates $\left(x_{i},y_{i},z_{i}\right)$ map the reference element to the physical element:

$$x\left(\xi ,\eta ,\zeta \right)=\sum_{i=1}^{8}x_{i}\phi_{i}\left(\xi ,\eta ,\zeta \right),\;y=\sum_{i=1}^{8}y_{i}\phi_{i},\;z=\sum_{i=1}^{8}z_{i}\phi_{i}.$$

The Jacobian matrix is:

$$J\left(\xi ,\eta ,\zeta \right)=\left[\begin{array}{ccc} \partial x/\partial \xi & \partial x/\partial \eta & \partial x/\partial \zeta \\ \partial y/\partial \xi & \partial y/\partial \eta & \partial y/\partial \zeta \\ \partial z/\partial \xi & \partial z/\partial \eta & \partial z/\partial \zeta \end{array}\right],$$

and dΩ=detJdξdηdζ. Global gradients are obtained by $\nabla \phi_{i}=J^{-1}{\left[\partial_{\xi }\phi_{i},\partial_{\eta }\phi_{i},\partial_{\zeta }\phi_{i}\right]}^{T}$.

#### Appendix A.3.3. Element Matrices: Thermal

For element *e*:

(A5) $$\begin{array}{cc} C_{ij}^{e} & =\int_{\Omega^{e}}\rho \left(T\right)\;c_{p}\left(T\right)\;\phi_{i}\phi_{j}\;d\Omega , \end{array}$$

(A6) $$\begin{array}{cc} K_{T,ij}^{e} & =\int_{\Omega^{e}}k\left(T\right)\;\nabla \phi_{i}\cdot \nabla \phi_{j}\;d\Omega , \end{array}$$

(A7) $$\begin{array}{cc} F_{q,i}^{e} & =\int_{\Omega^{e}}Q\left(x,t\right)\;\phi_{i}\;d\Omega +\int_{\partial \Omega_{q}^{e}}\bar{q}\;\phi_{i}\;d\Gamma . \end{array}$$

#### Appendix A.3.4. Element Matrices: Mechanical

Define strain-displacement matrix B(ξ) such that

$$\varepsilon =B\;U^{e}.$$

Then:

(A8) $$\begin{array}{cc} K^{e}\left(T\right) & =\int_{\Omega^{e}}B^{T}\;D\left(T\right)\;B\;d\Omega , \end{array}$$

(A9) $$\begin{array}{cc} F_{\mathrm{th}}^{e}\left(T\right) & =\int_{\Omega^{e}}B^{T}\;D\left(T\right)\;\varepsilon^{\mathrm{th}}\left(T\right)\;d\Omega , \end{array}$$

(A10) $$\begin{array}{cc} F_{\mathrm{body}}^{e} & =\int_{\Omega^{e}}N^{T}b\;d\Omega ,\;F_{\mathrm{ext}}^{e}=\int_{\Gamma_{t}^{e}}N^{T}\bar{t}\;d\Gamma . \end{array}$$

#### Appendix A.3.5. Constitutive Law and Thermal Strain

The local constitutive relation (Voigt form) is

$$\sigma =D\left(T\right)\left(\varepsilon -\varepsilon^{\mathrm{th}}\left(T\right)\right),$$

with isotropic thermal strain

$$\varepsilon^{\mathrm{th}}\left(T\right)=\alpha \left(T\right)\left(T-T_{\mathrm{ref}}\right)m,\;m={\left[1\;1\;1\;0\;0\;0\right]}^{T}.$$

#### Appendix A.3.6. Numerical Integration (Quadrature)

Use Gauss quadrature on the reference box. For trilinear elements a 2×2×2 rule (8 points) is the minimum:

$$\xi ,\eta ,\zeta \in \{\pm 1/\sqrt{3}\},\;w=1.$$

At each Gauss point evaluate:

$$J,\;\det J,\;J^{-1},\;\nabla \phi_{i},\;T\left(\mathrm{GP}\right),\;D\left(T\left(\mathrm{GP}\right)\right),\;\varepsilon^{\mathrm{th}}\left(T\left(\mathrm{GP}\right)\right).$$

#### Appendix A.3.7. Gauss-Sum Implementations

Element stiffness (thermal and mechanical) and loads are approximated by sums:

(A11) $$\begin{array}{cc} K^{e}\left(T\right) & \approx \sum_{g=1}^{n_{g}}w_{g}\;B^{T}\left(\xi_{g}\right)\;D\left(T_{g}\right)\;B\left(\xi_{g}\right)\;\det J_{g}, \end{array}$$

(A12) $$\begin{array}{cc} F_{\mathrm{th}}^{e}\left(T\right) & \approx \sum_{g=1}^{n_{g}}w_{g}\;B^{T}\left(\xi_{g}\right)\;D\left(T_{g}\right)\;\varepsilon^{\mathrm{th}}\left(T_{g}\right)\;\det J_{g}, \end{array}$$

(A13) $$\begin{array}{cc} C_{ij}^{e} & \approx \sum_{g=1}^{n_{g}}w_{g}\;\rho \left(T_{g}\right)c_{p}\left(T_{g}\right)\;\phi_{i}\left(\xi_{g}\right)\phi_{j}\left(\xi_{g}\right)\;\det J_{g}. \end{array}$$

Figure A2 illustrates the complete isoparametric mapping procedure for a representative hexahedral element. The transformation from the reference element in (ξ,η,ζ) coordinates to the physical element with nodal temperatures $T_{i}$ demonstrates how temperature-dependent material properties are evaluated at Gauss points. This element-level computation forms the foundation of the layer-by-layer simulation strategy, where localized laser heating produces steep thermal gradients that must be accurately captured through appropriate spatial discretization.

### Appendix A.4. Assembly, Boundary Conditions, and Solution

#### Appendix A.4.1. Assembly

Assemble element contributions into global arrays using assembly operator $A^{e}$:

$$K=\sum_{e}{\left(A^{e}\right)}^{T}K^{e}A^{e},\;C=\sum_{e}{\left(A^{e}\right)}^{T}C^{e}A^{e},\;F=\sum_{e}{\left(A^{e}\right)}^{T}F^{e}.$$

#### Appendix A.4.2. Boundary Conditions

##### Dirichlet BCs

Enforce displacement or temperature essential BCs by row/column elimination or Lagrange multipliers/penalty methods.

##### Neumann BCs

Add traction or flux contributions to the global load vector using surface quadrature on element faces.

#### Appendix A.4.3. Time Integration (Thermal)

Discretize (A3) in time with implicit backward Euler:

$$\left(\frac{C}{\Delta t}+K_{T}\right)T^{n+1}=\frac{C}{\Delta t}T^{n}+F_{q}^{\;n+1}.$$

Solve for $T^{n+1}$; update temperature-dependent properties and mechanical inputs.

#### Appendix A.4.4. Mechanical Solve

With updated temperature field compute $K(T^{n+1})$ and $F_{\mathrm{th}}\left(T^{n+1}\right)$. Solve quasi-static equilibrium:

$$K\left(T^{n+1}\right)\;U^{n+1}=F_{\mathrm{ext}}+F_{\mathrm{th}}\left(T^{n+1}\right).$$

### Appendix A.5. Coupling Strategies and Linearization

#### Appendix A.5.1. Staggered (Partitioned) Coupling

A practical approach per time step:

1. Solve thermal problem for $T^{n+1}$.
2. Assemble mechanical matrices using $T^{n+1}$ and solve for $U^{n+1}$.
3. If strong coupling: iterate thermal ↔ mechanical until residuals converge.

#### Appendix A.5.2. Monolithic Coupling and Newton Linearization

Define residual

$$R\left(u,T\right)=\left[\begin{array}{c} C\dot{T}+K_{T}T-F_{q}\\ K\left(T\right)U-F_{\mathrm{ext}}-F_{\mathrm{th}}\left(T\right) \end{array}\right].$$

Linearize for Newton updates (δU,δT). The coupled Jacobian requires blocks such as ∂K/∂T and $\partial F_{\mathrm{th}}/\partial T$, computed elementwise at Gauss points:

(A14) $$\begin{array}{cc} \frac{\partial K^{e}}{\partial T}{|}_{g} & =B^{T}\left(\xi_{g}\right)\frac{\partial D}{\partial T}\left(T_{g}\right)B\left(\xi_{g}\right)\det J_{g}, \end{array}$$

(A15) $$\begin{array}{cc} \frac{\partial F_{\mathrm{th}}^{e}}{\partial T}{|}_{g} & =B^{T}\left(\xi_{g}\right)\left[\frac{\partial D}{\partial T}\left(T_{g}\right)\varepsilon^{\mathrm{th}}\left(T_{g}\right)+D\left(T_{g}\right)\frac{\partial \varepsilon^{\mathrm{th}}}{\partial T}\left(T_{g}\right)\right]\det J_{g}. \end{array}$$

### Appendix A.6. Verification, Validation and Best Practices

- Patch test: Uniform temperature increment should produce the known rigid-body-free expansion (check reactions under constraints).
- Power check: Integrate heat source *Q* over Ω or exposed surface; confirm total absorbed power equals ηP (or corrected prefactor for volumetric models).
- Mesh and quadrature: refine mesh where thermal gradients are large; increase Gauss order if needed.
- Time-step control: adaptive time stepping recommended when strong transients occur; use CFL-like criteria for explicit schemes (if used).
- Material models: ensure smooth interpolation/extrapolation of $E\left(T\right),\nu \left(T\right),k\left(T\right),c_{p}\left(T\right)$ to avoid unphysical jumps.

### Appendix A.7. Algorithm: Layer-by-Layer Thermo–Mechanical Coupling

Below we present a compact algorithmic description that directly maps to the FE steps above. This is suitable for implementation in a research-grade FE code.

The layer activation strategy is visualized in Figure A3, which shows the progressive element activation mechanism during additive manufacturing. As the laser heat source traverses the scan path, elements transition from an inactive state ($f_{a}=10^{-6}$) to fully active ($f_{a}=1.0$), simulating the physical deposition process without costly remeshing. The Gaussian distribution of the heat source creates localized thermal gradients that drive residual stress generation and part distortion, phenomena that are captured through the coupled thermo-mechanical framework presented in Algorithm A1, Algorithm A2, Algorithm A3, Algorithm A4 and Algorithm A5.

**Algorithm A1** Layer-by-Layer Thermo–Mechanical Coupling in Additive Manufacturing.
1: **function** AdditiveManufacturing(CAD,M,MaterialDB,ProcessParams,N) 2: **Inputs:** CAD model Ω, mesh M, Material database: $\rho \left(T\right),c_{p}\left(T\right),k\left(T\right),\alpha \left(T\right),D\left(T\right)$; Process parameters: P,R,v,η,h; BCs and ICs. 3: **Outputs:** Temperature T(x,t), stress σ(x,t), strain ε(x,t). 4: Initialize $\Omega \leftarrow \Omega_{\mathrm{sub}}$, t←0, $T^{0}\leftarrow T_{\mathrm{amb}}1$, $U^{0}\leftarrow 0$. 5: **for** L=1 to $N_{\mathrm{layers}}$ **do** 6: Ω←ActivateLayer(Ω,L) 7: Results[*L*] ← SimulateLayer(Ω,L,MaterialDB,ProcessParams) 8: **end for** 9: **return** Results 10: **end function**

**Algorithm A2** Activate Layer.
1: **function** ActivateLayer(Ω,L) 2: Sprinkle fresh powder. 3: $\Omega \leftarrow \Omega \cup \Omega_{L}$ ▹ Mesh update/activation or element birth 4: **return** Ω 5: **end function**

**Algorithm A3** Simulate Layer (thermal + mechanical).
1: **function** SimulateLayer(Ω,L,MaterialDB,ProcessParams) 2: Compute layer time window $\left[t_{\mathrm{layer},\;\mathrm{start}},t_{\mathrm{layer},\;\mathrm{end}}\right]$ and choose Δt 3: $t_{\mathrm{layer},\;\mathrm{start}}=\left(L-1\right)\times t_{\mathrm{layer},\;\mathrm{duration}}$ 4: $t_{\mathrm{layer},\;\mathrm{end}}=L\times t_{\mathrm{layer},\mathrm{duration}}$ 5: **for** $t\leftarrow t_{\mathrm{layer},\;\mathrm{start}}:\Delta t:t_{\mathrm{layer},\;\mathrm{end}}$ **do** 6: T←ThermalSolver(Ω,MaterialDB,ProcessParams,t) 7: (σ,ε,U)←MechanicalSolver(Ω,MaterialDB,T) 8: Check convergence: if $∥T^{n+1}-T^{n}∥<\epsilon_{T}$ and $∥U^{n+1}-U^{n}∥<\epsilon_{U}$ then proceed; else reduce Δt and repeat. 9: Store {T,σ,ε,U} for this time step. 10: **end for** 11: Apply any cooling or post-layer processing as required 12: **return** {T,σ,ε,U} 13: **end function**

**Algorithm A4** ThermalSolver (per time step).
1: **function** ThermalSolver(Ω,MaterialDB,ProcessParams,t) 2: Update heat source position $x_{\mathrm{source}}\left(t\right)$ along scan path. 3: Compute applied heat flux. For a top-surface Gaussian distribution: $$q\left(x,y,z,t\right)=\frac{2\eta P}{\pi R^{2}}\exp\;\left(-\frac{2r^{2}}{R^{2}}\right),$$ where *P* is power, η absorption efficiency, *R* beam radius, and *r* radial distance from the source center. In the finite element framework, this flux contributes to the nodal heat input vector $F_{q}$ through surface or volumetric integration. 4: Interpolate/update material properties at Gauss points: $\rho \left(T\right),c_{p}\left(T\right),k\left(T\right)$. 5: Assemble thermal element matrices and load vector: $M,K_{T},F_{q}$ (using Gauss quadrature). 6: Solve backward Euler: $$\left(\frac{C}{\Delta t}+K_{T}\right)T^{n+1}=\frac{C}{\Delta t}T^{n}+F_{q}^{\;n+1}.$$ 7: **return** $T^{n+1}$ 8: **end function**

**Algorithm A5** MechanicalSolver (per time step).
1: **function** MechanicalSolver(Ω,MaterialDB,T) 2: Evaluate D(T) and thermal strain $\varepsilon^{\mathrm{th}}\left(T\right)$ at Gauss points. 3: Assemble mechanical stiffness $K\left(T\right)=\sum_{e}K^{e}\left(T\right)$ and thermal load $F_{\mathrm{th}}\left(T\right)=\sum_{e}F_{\mathrm{th}}^{e}\left(T\right)$ via Gauss quadrature. 4: Apply boundary conditions (prescribed displacements/tractions). 5: Solve quasi-static equilibrium: $$K\left(T\right)U=F_{\mathrm{ext}}+F_{\mathrm{th}}\left(T\right).$$ 6: Post-process stresses: $\sigma =D\left(T\right)[\varepsilon \left(U\right)-\varepsilon^{\mathrm{th}}\left(T\right)]$. 7: **return** (σ,ε,U) 8: **end function**

### Appendix A.8. Implementation Notes and Tips

- Element activation: use element birth or activation for newly deposited layers; ensure consistent mapping and DOF numbering.
- Adaptive time stepping: reduce Δt when convergence criteria fail or when heat source moves rapidly.
- Mesh resolution: refine near the melt pool/beam path; use anisotropic refinement if required.
- Material models: smoothly interpolate temperature-dependent functions (avoid discontinuities).
- Parallelization: thermal solve per time step is compute-heavy; parallel assembly/solve recommended.
- Verification: use manufactured solutions or compare with experimental transient thermography and residual stress measurements where possible.

### Appendix A.9. Illustrative Example: Single-Element Thermal Analysis

To demonstrate the practical application of the finite element formulation presented in Section 3.1, Section 3.2, Section 3.3, Section 3.4, Section 3.5, Section 3.6 and Section 3.7, we provide a detailed step-by-step calculation for a single hexahedral element subjected to laser heating. This worked example bridges the gap between abstract mathematical expressions and concrete numerical implementation, facilitating verification and validation of user implementations.

#### Appendix A.9.1. Problem Setup

Consider a single 8-node hexahedral element representing one layer of an AlSi10Mg part during selective laser sintering:

- Element geometry: 1×1×0.03 mm (length × width × height)
- Material properties at T=500 K:
  – Thermal conductivity: k=150 W/(m·K)
  – Density: ρ=2670 kg/m^3^
  – Specific heat: $c_{p}=900$ J/(kg·K)
  – Elastic modulus: E=60 GPa
  – Thermal expansion coefficient: $\alpha =23\times 10^{-6}$ K^−1^
- Boundary conditions:
  – Gaussian heat flux on top surface: $q_{0}=10^{6}$ W/m^2^
  – Beam radius: R=0.1 mm
  – Initial temperature: $T_{0}=473$ K (preheated substrate)

#### Appendix A.9.2. Step 1: Element Capacitance Matrix

The thermal capacitance matrix is computed using 2 × 2 × 2 Gauss quadrature as specified in Equation (13):

$$C_{ij}^{e}=\int_{\Omega^{e}}\rho c_{p}\varphi_{i}\varphi_{j}\;d\Omega \approx \sum_{g=1}^{8}w_{g}\rho c_{p}\varphi_{i}\left(\xi_{g}\right)\varphi_{j}\left(\xi_{g}\right)\left|\det J_{g}\right|$$

For the first diagonal entry (i=j=1) at Gauss point $\xi_{1}=\left(-1/\sqrt{3},-1/\sqrt{3},-1/\sqrt{3}\right)$:

$$\begin{array}{cc} \varphi_{1}\left(\xi_{1}\right) & =\frac{1}{8}{\left(1-1/\sqrt{3}\right)}^{3}\approx 0.0456\\ \left|\det J\right| & =\frac{\left(1\;\mathrm{mm})(1\;\mathrm{mm})(0.03\;\mathrm{mm}\right)}{8}=3.75\times 10^{-3}\;{\mathrm{mm}}^{3}=3.75\times 10^{-9}\;m^{3}\\ C_{11}^{e} & \approx \left(1.0\right)\left(2670\right)\left(900\right){\left(0.0456\right)}^{2}\left(3.75\times 10^{-9}\right)\approx 1.58\times 10^{-5}\;J/K \end{array}$$

Summing contributions from all eight Gauss points and assembling the full matrix yields:

$$C^{e}\approx 1.01\times 10^{-4}I_{8\times 8}\;J/K\;(\mathrm{diagonal}\;\mathrm{approximation})$$

#### Appendix A.9.3. Step 2: Element Conductivity Matrix

The conductivity matrix requires shape function gradients computed via the Jacobian (Equation (6)):

$$K_{T,ij}^{e}=\int_{\Omega^{e}}k\nabla \varphi_{i}\cdot \nabla \varphi_{j}\;d\Omega$$

For node 1, the shape function gradient in the reference element is:

$$\nabla_{\xi }\varphi_{1}=\frac{1}{8}\left[\begin{array}{c} -(1+\eta )(1+\zeta )\\ -(1+\xi )(1+\zeta )\\ -(1+\xi )(1+\eta ) \end{array}\right]$$

The Jacobian matrix for this rectangular element is constant:

$$J=\left[\begin{array}{ccc} 0.5 & 0 & 0\\ 0 & 0.5 & 0\\ 0 & 0 & 0.015 \end{array}\right]\;\mathrm{mm},\;J^{-1}=\left[\begin{array}{ccc} 2 & 0 & 0\\ 0 & 2 & 0\\ 0 & 0 & 66.67 \end{array}\right]\;{\mathrm{mm}}^{-1}$$

Computing the physical gradient and evaluating the integral (see Figure A2 for visualization):

$$K_{T,11}^{e}\approx 1.69\times 10^{-3}\;W/K$$

#### Appendix A.9.4. Step 3: Thermal Load Vector

The surface heat flux contribution on the top face (z=0.03 mm) is evaluated using surface quadrature (Equation (7)):

$$F_{q,i}^{e}=\int_{S_{top}}q_{0}\exp\left(-\frac{r^{2}}{R^{2}}\right)\varphi_{i}\;dS$$

Assuming the element center coincides with the beam axis and using 2 × 2 surface quadrature:

$$\begin{array}{cc} r(\xi ,\eta ) & =\sqrt{{\left(x\left(\xi ,\eta \right)-x_{beam}\right)}^{2}+{\left(y\left(\xi ,\eta \right)-y_{beam}\right)}^{2}}\approx 0\\ F_{q,1}^{e} & \approx \frac{1}{4}\times \left(1\times 1\times 10^{-6}\right)\times 10^{6}\times 0.0456\times 1.0\approx 0.125\times 10^{-4}\;W \end{array}$$

For all eight nodes on the top surface, the total heat input is:

$$\sum_{i=1}^{8}F_{q,i}^{e}\approx 1.0\times 10^{-4}\;W$$

This value should be verified against the analytical integral of the Gaussian distribution to ensure energy conservation.

#### Appendix A.9.5. Step 4: Time Integration

Using the backward Euler scheme (implicit first-order) with time step $\Delta t=10^{-4}$ s:

$$\left(\frac{C^{e}}{\Delta t}+K_{T}^{e}\right)T^{n+1}=\frac{C^{e}}{\Delta t}T^{n}+F_{q}^{e}$$

For the first time step with $T^{0}=4731$ K:

$$\left(\frac{1.01\times 10^{-4}}{10^{-4}}+1.69\times 10^{-3}\right)T^{1}\approx 1.01\times T^{0}+F_{q}^{e}$$

Solving this 8×8 system yields temperature increments of approximately 0.1–0.3 K at nodes on the top surface, consistent with the rapid heating expected during laser exposure.

#### Appendix A.9.6. Step 5: Mechanical Response

Once the temperature field is established, the mechanical stiffness matrix and thermal load vector are computed following Equations (8) and (9). For this element at average temperature $\bar{T}=473.2$ K:

$$\begin{array}{cc} K^{e}\left(T\right) & =\int_{\Omega^{e}}B^{T}D\left(T\right)B\;d\Omega \approx 5.4\times 10^{3}\;N/\mathrm{mm}\;(\mathrm{representative}\;\mathrm{entry})\\ F_{th}^{e}\left(T\right) & =\int_{\Omega^{e}}B^{T}D\left(T\right)\varepsilon_{th}\left(T\right)\;d\Omega \end{array}$$

where thermal strain is:

$$\varepsilon_{th}=\alpha \left(\bar{T}-T_{ref}\right){\left[\begin{array}{cccccc} 1 & 1 & 1 & 0 & 0 & 0 \end{array}\right]}^{T}=23\times 10^{-6}\times 0.2\approx 4.6\times 10^{-6}$$

This thermal strain generates compressive stresses upon cooling, contributing to the residual stress field observed in fabricated parts.

#### Appendix A.9.7. Verification and Scaling

This single-element calculation demonstrates the complete workflow described in Algorithm A4 and Algorithm A5. When extended to the full mesh (approximately 400,000 elements as reported in Section 5.3) and integrated over 167 layers with progressive element activation (Figure A3), this methodology produces the thermo-mechanical response shown in Figure 6, Figure 7 and Figure 8 of Section 5.

Key verification steps:

1. Energy balance: Total heat input should equal $\eta P\times t_{exposure}$
2. Temperature bounds: $T_{min}=T_{substrate}$, $T_{max}<T_{vaporization}$
3. Mechanical equilibrium: Sum of nodal forces ≈0 (within solver tolerance)
4. Thermal strain recovery: $\varepsilon_{th}$ should produce expected thermal expansion

This worked example provides a concrete reference for users implementing the OpenAM-SimCCX framework, enabling direct verification of element-level calculations before proceeding to full-scale simulations.

### Appendix A.10. Expanded Description of the Coupled Thermo–Mechanical Analysis in CalculiX

The coupled thermo–mechanical simulations were performed using CalculiX, an open-source finite element solver. Input files were generated using a combination of Prepomax and Python scripts, however for academic usage Cubit-CalculiX add-on can help to automate mesh creation, material assignment, and load definitions.

Thermo-mechanical finite element simulation of additive manufacturing (AM) and welding requires the consistent integration of heat input, boundary fluxes, element formulation, and thermal flux recovery. Within the finite element solver CalculiX, this workflow is realized through four core user-defined Fortran subroutines: weld_deposit.f, dflux.f, e_c3d_th.f, and resultstherm.f. These routines collectively define the physics of the coupled thermal–mechanical system, beginning with the specification of heat sources and boundary conditions, followed by the formulation of element-level matrices and vectors, and concluding with the recovery of thermal fluxes and material state updates. Together, they form the computational backbone of the simulation framework, enabling accurate representation of heat input, conduction, boundary interactions, and flux recovery in AM and welding processes.

### Appendix A.11. Element Activation and Material Deposition (weld_deposit.f)

The weld_deposit subroutine governs the sequential activation of elements to simulate material deposition in layer-by-layer manufacturing. This is achieved through a geometric activation criterion based on a predefined welding trajectory and process parameters (e.g., layer thickness, scan speed, and scan pattern). The method avoids costly remeshing by employing the “quiet element” technique, where inactive elements retain negligible stiffness and conductivity until activated.

Activation Strategy: The subroutine activates elements progressively as the heat source traverses the deposition path. The welding distance traveled at time *t* is given by

(A16) $$d_{\mathrm{weld}}=v\cdot \left(t-t_{\mathrm{start}}\right),$$

where *v* is the welding speed and $t_{\mathrm{start}}$ the initial activation time for the current trajectory.

Activation Factor: Material state is controlled via an activation factor $f_{a}\left(x,t\right)$ assigned at each integration point:

(A17) $$f_{a}\left(x,t\right)=\left\{\begin{array}{cc} 1.0 & \mathrm{if}\;\mathrm{material}\;\mathrm{is}\;\mathrm{deposited}\;\mathrm{and}\;\mathrm{active},\\ 10^{-6} & \mathrm{if}\;\mathrm{material}\;\mathrm{is}\;\mathrm{inactive}\;(\mathrm{powder}\;\mathrm{or}\;\mathrm{void}). \end{array}\right.$$

The factor $f_{a}$ scales material properties such as conductivity and density, enabling a smooth transition from inactive to solid states.

Geometric Implementation: The global coordinates of an integration point (X,Y,Z) are transformed into the local coordinate system of the moving heat source defined by position $\left(X_{0},Y_{0},Z_{0}\right)$ and orientation (α,β,γ):

(A18) $$\left[\begin{array}{c} X^{\prime }\;Y^{\prime }\;Z^{\prime } \end{array}\right]=\left[R_{z}\left(\gamma \right)\right]\left[R_{y}\left(\beta \right)\right]\left[R_{x}\left(\alpha \right)\right]\left[\begin{array}{c} X-X_{0}\;Y-Y_{0}\;Z-Z_{0} \end{array}\right].$$

Elements are activated when a sufficient number of their integration points fall within the deposition zone, ensuring numerical stability.

Time-Based Layer Determination:

The current active layer is determined based on the elapsed manufacturing time:

(A19) $$\begin{array}{cc} t_{weld} & =t_{total}-t_{start} \end{array}$$

(A20) $$\begin{array}{cc} n_{layer} & =1+\sum_{i=1}^{n_{max}}1_{\left[t_{weld}>i\cdot t_{layer}\right]} \end{array}$$

(A21) $$\begin{array}{cc} t_{adjusted} & =t_{weld}-\left(n_{layer}-1\right)\times t_{layer} \end{array}$$

where $1_{\left[\cdot \right]}$ is the indicator function and $n_{max}=\left\lfloor h_{total}/h_{layer}\right\rfloor -1$.

Raster Trajectory Generation

The material deposition follows a bidirectional raster scanning pattern within each layer. The trajectory coordinates are generated using Algorithm A6:

**Algorithm A6** Bidirectional Raster Pattern Generation.
1: Initialize: cot(1,1)=0, cot(2,1)=0.25, $cot\left(3,1\right)=n_{layer}\times h_{layer}$ 2: Set: right=1, $n_{nodes}=2\times w_{total}/r_{res}$ 3: **for** i=2 to $n_{nodes}$ step 2 **do** 4: **if** right>0 **then** 5: $cot\left(1,i\right)=L_{scan}$ ▹ Right-to-left scan 6: cot(2,i)=cot(2,i−1) 7: right=0 8: **else** 9: cot(1,i)=0.0 ▹ Left-to-right scan 10: cot(2,i)=cot(2,i−1) 11: right=1 12: **end if** 13: $cot\left(3,i\right)=n_{layer}\times h_{layer}$ 14: cot(1,i+1)=cot(1,i) 15: $cot\left(2,i+1\right)=cot\left(2,i\right)+r_{res}$ ▹ Advance to next row 16: cot(3,i+1)=cot(3,i) 17: **end for**

### Appendix A.12. Trajectory Distance Calculation

The cumulative distance along the deposition path is calculated for position tracking:

(A22) $$\begin{array}{cc} d_{i} & =\sqrt{{\left(x_{i+1}-x_{i}\right)}^{2}+{\left(y_{i+1}-y_{i}\right)}^{2}+{\left(z_{i+1}-z_{i}\right)}^{2}} \end{array}$$

(A23) $$\begin{array}{cc} d_{total} & =\sum_{i=1}^{n_{segments}}d_{i} \end{array}$$

The current deposition distance is:

(A24) $$d_{current}=v_{speed}\times t_{adjusted}$$

Raster Scanning Logic: The subroutine supports alternating scan directions per layer to mitigate heat accumulation. For a given layer of thickness layer_th, the activation logic distinguishes between:

- Above current layer: elements remain inactive, $f_{a}=10^{-6}$,
- Deposited or previous layers: elements are fully active, $f_{a}=1.0$,
- Current deposition zone: elements activated once the heat source passes.

Role in Simulation: By activating elements sequentially along the welding trajectory, the weld_deposit subroutine progressively evolves the computational domain. This approach captures the spatial and temporal characteristics of material deposition while avoiding remeshing, making it essential for efficient thermo-mechanical simulations of additive manufacturing processes.

### Appendix A.13. Heat Source Definition (dflux.f)

The dflux subroutine defines the volumetric heat input from a moving energy source, primarily tailored for laser welding and additive manufacturing. The formulation adapts the Goldak double-ellipsoid model into a conical Gaussian distribution to realistically capture laser penetration effects.

Mathematical Formulation: The volumetric heat flux is expressed as:

(A25) $$q\left(x,y,z\right)=Q_{0}\exp\left(-\frac{r^{2}}{R_{0}{\left(z\right)}^{2}}\right),$$

where the effective intensity is defined by

$$Q_{0}=\eta P,$$

with η the absorption efficiency and *P* the laser power. The depth-dependent radius follows a linear variation:

$$R_{0}\left(z\right)=R_{e}-\left(R_{e}-R_{i}\right)\cdot \frac{\left(Z_{e}-z\right)}{\left(Z_{e}-Z_{i}\right)},$$

where $R_{e}$ and $R_{i}$ are the top and bottom radii of the conical heat source, and $Z_{e},Z_{i}$ denote the top and penetration depth coordinates. The radial distance in the local coordinate system is given by $r^{2}=x_{l}^{2}+y_{l}^{2}$.

Coordinate Transformation: To compute *r*, global integration point coordinates (X,Y,Z) are transformed to the moving heat source’s local system using rotation matrices:

(A26) $$\left[\begin{array}{c} x_{l}\\ y_{l}\\ z_{l} \end{array}\right]=\left[R_{z}\left(\gamma \right)\right]\left[R_{y}\left(\beta \right)\right]\left[R_{x}\left(\alpha \right)\right]\left[\begin{array}{c} X-X_{0}\\ Y-Y_{0}\\ Z-Z_{0} \end{array}\right],$$

where $\left(X_{0},Y_{0},Z_{0}\right)$ and (α,β,γ) denote the source position and orientation. The current position of the heat source along the trajectory is determined by:

(A27) $$\left\{X_{0},Y_{0},Z_{0}\right\}=\left\{cot\left(1,i\right),cot\left(2,i\right),cot\left(3,i\right)\right\}\;\mathrm{where}\;\sum_{j=1}^{i-1}d_{j}\leq d_{current}<\sum_{j=1}^{i}d_{j}$$

The orientation angles for coordinate transformation are calculated as:

Gamma rotation (about Z-axis):

(A28) $$\gamma =\left\{\begin{array}{cc} \frac{\pi }{2} & \mathrm{if}\;\Delta y_{i,i+1}=0\\ 0 & \mathrm{if}\;\Delta x_{i,i+1}=0\\ \frac{\pi }{2}-\arctan\left(\frac{\Delta y_{i,i+1}}{\Delta x_{i,i+1}}\right) & \mathrm{otherwise} \end{array}\right.$$

Beta rotation (about Y-axis):

(A29) $$\beta =\left\{\begin{array}{cc} \frac{\pi }{2} & \mathrm{if}\;\Delta z_{i,i+1}=0\\ 0 & \mathrm{if}\;\Delta x_{i,i+1}=0\\ \frac{\pi }{2}-\arctan\left(\frac{\Delta z_{i,i+1}}{\Delta x_{i,i+1}}\right) & \mathrm{otherwise} \end{array}\right.$$

Alpha rotation (about X-axis):

(A30) $$\alpha =\left\{\begin{array}{cc} \frac{\pi }{2} & \mathrm{if}\;\Delta y_{i,i+1}=0\\ 0 & \mathrm{if}\;\Delta z_{i,i+1}=0\\ \frac{\pi }{2}-\arctan\left(\frac{\Delta z_{i,i+1}}{\Delta y_{i,i+1}}\right) & \mathrm{otherwise} \end{array}\right.$$

Layer progression is synchronized with the welding trajectory defined in weld_deposit, ensuring consistency in multi-layer additive processes.

Role in Simulation: The dflux subroutine governs the applied thermal load, enabling the thermal field to evolve under a physically realistic moving laser source. Its depth-dependent Gaussian profile captures the localized nature of heat deposition and is essential for accurately modeling melt pool dynamics and thermal histories in welding and additive manufacturing.

### Appendix A.14. Element Matrix Assembly (e_c3d_th.f)

The e_c3d_th subroutine is the core routine for three-dimensional thermal elements, responsible for forming the element-level matrices for both thermal and mechanical behavior. It computes conductivity, capacitance, mechanical stiffness, and thermal-mechanical coupling contributions through numerical integration, while also handling progressive material activation.

Functions:

- Assemble mechanical stiffness matrices: elastic ($K_{uu,e}$) and geometric/stress stiffness ($K_{\sigma ,e}$)
- Assemble thermal matrices: conductivity ($K_{TT,e}$) and capacity ($C_{e}$)
- Compute thermal-mechanical coupling contributions via thermal expansion ($f_{th,e}$ or $K_{uT,e}$)
- Integrate temperature-dependent material properties over element volumes
- Implement element activation/deactivation for progressive deposition

Mathematical Formulation:

The coupled element equations can be written in partitioned matrix form:

(A31) $$\left[\begin{array}{cc} M_{e} & 0\\ 0 & C_{e} \end{array}\right]\left[\begin{array}{c} {\ddot{u}}_{e}\\ {\dot{T}}_{e} \end{array}\right]+\left[\begin{array}{cc} K_{uu,e}+K_{\sigma ,e} & K_{uT,e}\\ K_{Tu,e} & K_{TT,e} \end{array}\right]\left[\begin{array}{c} u_{e}\\ T_{e} \end{array}\right]=\left[\begin{array}{c} f_{u,e}\\ f_{q,e} \end{array}\right].$$

Here:

- $u_{e}$: element displacement DOFs
- $T_{e}$: element temperature DOFs
- $M_{e}$: mass matrix (mechanical inertia)
- $C_{e}$: thermal capacity matrix
- $K_{uu,e}$: mechanical stiffness
- $K_{\sigma ,e}$: geometric (stress) stiffness
- $K_{TT,e}$: thermal conductivity (diffusion)
- $K_{uT,e}$: mechanical response to temperature (thermal expansion)
- $K_{Tu,e}$: temperature response to mechanical deformation (often zero in small-strain)
- $f_{u,e}$: mechanical loads (body forces, eigenstresses, thermal forces)
- $f_{q,e}$: thermal loads (heat fluxes and internal sources)

Element Integrals:

(A32) $$\begin{array}{cccc} K_{uu,e} & =\int_{V^{e}}B^{T}D\;B\;dV,\;\;\;\;\;\;\;\; & K_{\sigma ,e} & =\int_{V^{e}}B_{\sigma }^{T}\sigma \;B_{\sigma }\;dV, \end{array}$$

(A33) $$\begin{array}{cccc} \;\;K_{TT,e} & =\int_{V^{e}}{\left(\nabla N\right)}^{T}\;k\;\left(\nabla N\right)\;dV,\;\;\;\;\;\; & C_{e} & =\int_{V^{e}}\left(\rho c_{p}\right)N^{T}N\;dV, \end{array}$$

(A34) $$\begin{array}{cccc} \;\;f_{th,e} & =\int_{V^{e}}B^{T}D\alpha \;\Delta T\;dV,\;\;\;\;\;\; & K_{uT,e} & =-\int_{V^{e}}B^{T}D\alpha \;N\;dV, \end{array}$$

(A35) $$\begin{array}{cc} f_{q,e} & =\int_{S^{e}}N^{T}q\;dS+\int_{V^{e}}N^{T}Q\;dV.\;\;\;\;\;\;\;\;\;\;\;\; \end{array}$$

In these integrals:

- B=∇N, gradient of displacement shape functions
- N, shape function matrix for temperature
- k, thermal conductivity
- ρ, density; $c_{p}$, specific heat
- D, elastic stiffness tensor
- α, thermal expansion tensor
- $f_{a}$, activation factor applied to scale material properties (inactive elements: $f_{a}\ll 1$, fully active: $f_{a}=1$)

Simulation Role:

e_c3d_th.f provides the finite element contributions necessary for coupled thermo-mechanical analysis. Thermal expansion is incorporated as equivalent nodal forces or a stiffness column, enabling accurate representation of progressive deposition without remeshing. Thermal and mechanical subproblems are solved sequentially or in a coupled manner depending on the solution scheme.

### Appendix A.15. Thermal Results and State Variable Update (resultstherm.f)

The resultstherm subroutine is executed after each thermal increment, once the nodal temperatures are obtained. Its role is twofold: (i) to compute the heat flux at integration points, and (ii) to update state variables that govern material activation and temperature-dependent behavior.

Heat Flux Calculation: Heat fluxes are computed using Fourier’s law, with material properties scaled by the activation factor $f_{a}$:

(A36) $$q=-f_{a}\cdot k\cdot \nabla T,$$

where the temperature gradient is obtained from nodal values via the shape function derivatives:

(A37) $$\frac{\partial T}{\partial x_{i}}=\sum_{j=1}^{n_{node}}\frac{\partial N_{j}}{\partial x_{i}}T_{j}.$$

State Variable Management: The subroutine maintains consistency across increments by updating:

- Activation factors ($f_{a}$) for progressive material deposition,
- Temperature-dependent material properties ($k,\rho ,c_{p}$),
- Activation flags for elements, ensuring readiness for the next increment.

Coupling with weld_deposit: For integration points that remain inactive ($f_{a}<1$), resultstherm calls weld_deposit to evaluate whether the point should be activated in the current increment based on the updated time and heat source trajectory. This ensures synchronization between deposition logic and thermal response.

Role in Simulation: The resultstherm subroutine bridges the thermal solution and the evolving material domain. By computing derived quantities such as heat flux and ensuring consistent state updates, it provides the necessary information for both post-processing and for guiding subsequent increments in thermo–mechanical simulations.

#### Workflow Integration

The four subroutines operate sequentially within each time step as shown in Figure 2:

1. weld_deposit: applies localized Gaussian heat input $f_{a}$.
2. dflux: imposes additional boundary fluxes q=f(x,y,z,t,T).
3. e_c3d_th: assembles $K^{e}$, $M^{e}$, and $F^{e}$, integrating heat input and boundary conditions.
4. resultstherm: recovers fluxes and updates tangent matrices $D_{th}$.

This workflow captures thermal gradients, melt pool evolution, and residual stress formation, essential for predicting distortions, microstructural changes, and defects.

These four core subroutines -weld_deposit.f, dflux.f, e_c3d_th.f, and resultstherm.f, inputs, outputs, and roles illustrated in Table A1-form a coherent, integrated finite element workflow for simulating coupled thermal–mechanical behavior in AM and welding. Sequential execution allows accurate prediction of temperature fields, melt pool dynamics, residual stress evolution, and structural distortions, providing a comprehensive framework for modeling the complex thermomechanical processes inherent in additive manufacturing and welding.

**Table A1:** Summary of core Fortran subroutines and their respective roles and inputs/outputs within the Directed Energy Deposition (DED) simulation framework.

Subroutine	Primary Role	Key Inputs → Outputs
weld_deposit	Geometric Activation	Time, Coordinates → Activation factor $f_{a}$
dflux	Heat Source Definition	Time, Coordinates, Power → Heat flux *q*
resultstherm	State Update & Flux Calculation	Nodal Temperatures, Previous State → Heat flux q, Updated State
e_c3d_th	Element Matrix Assembly	Activation factor $f_{a}$, Material Properties → Element Matrices $K^{e},C^{e}$
